# Mathematical study of neural feedback roles in small target motion detection

**DOI:** 10.3389/fnbot.2022.984430

**Published:** 2022-09-20

**Authors:** Jun Ling, Hongxin Wang, Mingshuo Xu, Hao Chen, Haiyang Li, Jigen Peng

**Affiliations:** ^1^School of Mathematics and Information Science, Guangzhou University, Guangzhou, China; ^2^Machine Life and Intelligence Research Center, Guangzhou University, Guangzhou, China; ^3^Computational Intelligence Lab (CIL), School of Computer Science, University of Lincoln, Lincoln, United Kingdom

**Keywords:** visual system modeling, small target motion detection, feedback mechanism, existence of solutions, fixed point iteration

## Abstract

Building an efficient and reliable small target motion detection visual system is challenging for artificial intelligence robotics because a small target only occupies few pixels and hardly displays visual features in images. Biological visual systems that have evolved over millions of years could be ideal templates for designing artificial visual systems. Insects benefit from a class of specialized neurons, called small target motion detectors (STMDs), which endow them with an excellent ability to detect small moving targets against a cluttered dynamic environment. Some bio-inspired models featured in feed-forward information processing architectures have been proposed to imitate the functions of the STMD neurons. However, feedback, a crucial mechanism for visual system regulation, has not been investigated deeply in the STMD-based neural circuits and its roles in small target motion detection remain unclear. In this paper, we propose a time-delay feedback STMD model for small target motion detection in complex backgrounds. The main contributions of this study are as follows. First, a feedback pathway is designed by transmitting information from output-layer neurons to lower-layer interneurons in the STMD pathway and the role of the feedback is analyzed from the view of mathematical analysis. Second, to estimate the feedback constant, the existence and uniqueness of solutions for nonlinear dynamical systems formed by feedback loop are analyzed *via* Schauder's fixed point theorem and contraction mapping theorem. Finally, an iterative algorithm is designed to solve the nonlinear problem and the performance of the proposed model is tested by experiments. Experimental results demonstrate that the feedback is able to weaken background false positives while maintaining a minor effect on small targets. It outperforms existing STMD-based models regarding the accuracy of fast-moving small target detection in visual clutter. The proposed feedback approach could inspire the relevant modeling of robust motion perception robotics visual systems.

## 1. Introduction

Small target motion detection is one of the most important problems in computer vision, and it has been widely applied in underwater robot vision (Xu et al., 2018), security monitoring (Escobar-Alvarez et al., 2019), unmanned driving (Li et al., 2017), military interception (Bosquet et al., 2018), etc. However, a small target occupies few pixels in an image so that it hardly displays physical features. Moreover, the complex dynamic environment always contains a great number of small-target-like features (such as leaves, flowers, and shadows), and there often exists ego-motion during sampling *via* camera. These phenomena mentioned above will bring great difficulties to small target motion detection.

The traditional computer vision methods for objects detection, including background subtraction (Saleemi and Shah, 2013), temporal differencing (Shuigen et al., 2009; Javed et al., 2018), and optical flow (Fortun et al., 2015). These conventional methods achieve sufficiently good performance in detecting large objects (such as pedestrians, cars, and vehicles) in static background. However, their detection performance to detect a small target with the dynamic background will decrease significantly due to the small target always concealing in background clutter and hardly displaying visual features. In addition, some machine learning methods, such as convolutional neural networks (Redmon et al., 2016) and support vector machines (Tang et al., 2017) can be used for object detection. These machine learning methods perform well in detecting objects with high resolution, clear appearance, and structure from which the discriminative features can be learned. However, they may fail to detect small objects with only one or a few pixels in size, since rich representations are difficult to learn from their poor-quality appearance and structure.

In nature, insects, such as dragonfly, hoverfly, and drosophila, display exquisite sensitivity to small target motion and are able to pursue potential mates and small prey with success rates greater than 97% in complex dynamic environments (Mischiati et al., 2015). Electrophysiological experiments have identified a class of special neurons in the brain of insects, called Small Target Motion Detectors (STMDs) (Nordström and O'Carroll, 2006; Nordstrom and O'Carroll, 2009; Barnett et al., 2007; Keleş and Frye, 2017), which make insects sensitive to small target motion. More precisely, the STMDs respond strongly to small moving targets subtending 1° ~ 3° of the visual field, but weakly to larger objects subtending more than 10° (Nordström et al., 2006) or wide-field grating stimuli. In addition, STMD neurons also respond robustly to small targets moving in the complex dynamic background. These distinctive functions of STMDs provide reliable support for the design of small target motion detection models.

Inspired by the insect's vision system, some models have been proposed to imitate the functions of STMDs. For example, as pioneering work, a computational model, called Elementary STMD (ESTMD), was designed to implement the size selectivity of the STMDs and detect the moving small target (Wiederman et al., 2008). The ESTMD displays strong responses to the presence of small moving targets, while weak or no responses to a large moving object. In order to estimate motion directions, the Elementary Motion Detector designed by Hassenstein and Reichardt (Hassenstein and Reichardt, 1956) was incorporated into ESTMD (Wiedermann and O'Carroll, 2013a,b), generating two new models, named EMD-ESTMD and ESTMD-EMD, respectively. Recently, Wang et al. developed a directionally selective STMD (DSTMD) (Wang et al., 2018), which makes use of correlation mechanism of two locations to detect positions and motion directions of small moving targets. On the base of DSTMD, Wang et al. (2019) exploited a direction contrast pathway to filter out fake features, where the resulted model is called STMD-plus. These models mentioned above process information in a feed-forward manner to detect small target motion. Despite the success of these feed-forward models in small target motion detection, the detection performances of these models in the complex dynamic background are unsatisfying and their detection results contain a number of background false positives.

The feedback mechanism plays an important role in modulating visual stimuli in animals' visual systems (Lamme et al., 1998; Bastos et al., 2015; Clarke and Maler, 2017), and it can potentiate the abilities of visual neurons to detect the motion of targets in a complex dynamic environment (Klink et al., 2017; Mohsenzadeh et al., 2018; Nurminen et al., 2018). Further biological research studies have also revealed feedback loops in insect visual systems. For instance, insects used closed-loop control in their brains to distinguish whether or not the changes in the environment are caused by their own behavior (Paulk et al., 2015). Insects selectively responded to salient visual stimuli *via* a close-loop in the brain (Paulk et al., 2014), which guided behavioral choices made by these insects. The feedback connection was discovered in the binocular stereopsis of praying mantis (Rosner et al., 2019), which calculated the distances from disparities between the two retinal images *via* feedback to trigger a raptorial strike of their forelegs when prey is within reach. In recent years, feedback mechanism has been proven to effectively improve model performance in many studies, including medical image segmentation (Soker, 2016) and object recognition (Wang and Huang, 2015). However, their connection patterns and functional roles in small target motion detection pathway still remain unclear. Based on the feedback mechanism, Wang et al. (2018) developed a feedback STMD model (Feedback STMD) for small target motion detection. The Feedback STMD was modeled by transmitting the output layer information to medulla layer neurons to filter out fake features. Although the Feedback STMD performed well in detecting the small target, its detection results contain a number of background false positives in complex dynamic backgrounds. In addition, the feedback model only verified the effectiveness *via* experiments, and the feedback constant was chosen by empiric rule, lacking mathematical proof analysis.

In this paper, we proposed a new time-delay feedback model, called FSTMD, for detecting small target motion in complex dynamic backgrounds. The FSTMD forms a nonlinear dynamic system by propagating the output of higher-layer neurons to the lower-layer neurons for weakening background fake features. The main contributions of this paper can be summarized as follows:

**1)**. We develop a time-delay feedback STMD model for small target motion detection by transmitting the STMD neurons outputs to the lamina neurons to weaken the background false positives.
**2)**. The functional role of the feedback is revealed by comparing the outputs of with and without feedback to different velocities.
**3)**. To estimate the strength of the feedback, we prove the existence and uniqueness of the solutions to the nonlinear dynamic system *via* Schauder's fixed point theorem and contraction mapping theorem.
**4)**. We design an iterative algorithm to find the approximate solution of a nonlinear system and verify the effectiveness of the algorithm *via* experiments. The results of our experiments demonstrate that our proposed model is unable to improve the detection performance in detecting small target motions on a complex background.

The article is structured as follows. In Section 2, some related works are introduced. In Section 3, the FSTMD model and the working mechanism of feedback are introduced in detail. In Section 4, the existence and uniqueness of the solutions to the nonlinear dynamic system are proved and an iterative algorithm is proposed. In Section 5, experimental results demonstrate the advantage of the proposed algorithm over other STMD-based algorithms in detecting motion of small targets. Finally, some conclusions are given in Section 6 and the proofs of the theorems are provided in Appendix.

## 2. Related work

In this section, some related works are introduced, mainly including some STMD-based models, feedback mechanism, and infrared small target detection.

### 2.1. The STMD-based models

Small target motion detectors are a special kind of motion-sensitive neurons, which respond strongly to small target motion even in complex dynamic backgrounds. Motivated by the superior properties of the STMD neurons, some STMD-based models have been developed for small target motion detection. For instance, as a pioneer, Wiederman et al. (2008) first proposed an Elementary Small Target Motion Detector (ESTMD) which well matches to the size selectivity of the STMDs and can detect the presence of small moving targets. However, the ESTMD model is not directionally selective and cannot estimate the direction of motion. In order to model the directional selectivity of STMDs, Wiedermann and O'Carroll (2013b) developed two mixed models, including EMD-ESTMD and ESTMD-EMD, which are designed by the Elementary Motion Detector (Hassenstein and Reichardt, 1956) combined with ESTMD (Wiedermann and O'Carroll, 2013a,b). However, the motion direction is only divided into four directions, i.e., right/up and left/down. Recently, Wang et al. (2018) developed a directionally selective STMD (DSTMD), which makes use of the correlation mechanism of two locations to detect positions and estimate motion directions of small moving targets systematically. On the base of DSTMD, Wang et al. (2019) exploited a direction contrast pathway to filter out most of the fake features. These models mentioned above all process visual information in a feedforward manner. However, feedback is a common regulatory mechanism in biology and has been not investigated in the STMD pathway.

### 2.2. Feedback mechanism

Feedback is a fundamental mechanism which regulates visual signals in the biological visual system. It refers to the process of returning the output of the system to the input and changing the input in some way and affecting the system function. The feedback mechanism has been applied in the field of artificial intelligence to achieve higher performance. For example, Carreira et al. (2016) proposed a framework to pose estimation. It enhances the expressive ability of the hierarchical feature extractors *via* top-down feedback and shows excellent performance in articulated pose estimation. Cao et al. (Cao et al., 2018) developed a Feedback CNN model, which could implement the selectivity mechanism of neuron activation and localize and segment the interested objects accurately in images. Zhang et al. (2018) introduced multi-path recurrent feedback to enhance salient target detection. It enhanced effective feature learning by introducing multi-path recurrent feedback to transfer global semantic information from the top-level convolution layer to the shallower layer and performed favorably against the state-of-the-art approaches. In addition, feedback has also been used extensively in the nonlinear dynamic system to pursue the stability problem of nonlinear systems and design feedback controller over the past decade (Liu and Tong, 2015; Sahoo et al., 2015; Brunton et al., 2016). Although feedback mechanisms have achieved great success in many fields, the feedback connection mode and function to STMD neural pathways still remain unclear.

### 2.3. Infrared small target detection

There are many conventional computer vision methods for objects detection, including background subtraction (Saleemi and Shah, 2013), temporal differencing (Shuigen et al., 2009; Javed et al., 2018), and optical flow (Fortun et al., 2015). Most of conventional computer vision methods achieve sufficiently good performance in detecting large objects, such as pedestrians, cars, and vehicles. However, these methods are powerless to detect small targets. The reason is that the small targets hardly show features such as shape, color, and structure. In addition, the current small target movement detection mainly focuses on infrared images. For example, an infrared image patch-image model was proposed by formulating an optimization problem of recovering low-rank, which achieved superior performance for different target sizes and signal-to-clutter ratio values (Gao et al., 2013). Bai et al. proposed the derivative entropy-based contrast measure for small target detection under various complex background clutters. It applied the derivative entropy-based contrast measure to enhance the infrared small target detection and suppress background clutter (Bai and Bi, 2018). Deng et al. applied a special ring Top-Hat transformation to suppress the complex background and developed a novel local entropy for capturing local features and target enhancement to enhance the infrared small target detection (Deng et al., 2021). These methods have performed well to detect small targets on infrared images, which are heavily dependent on temperature differences between the background and small targets. However, they are impotent for small target motion detection on natural cluttered backgrounds.

## 3. Formulation of the model and the working mechanism of feedback

**Figure 1:**
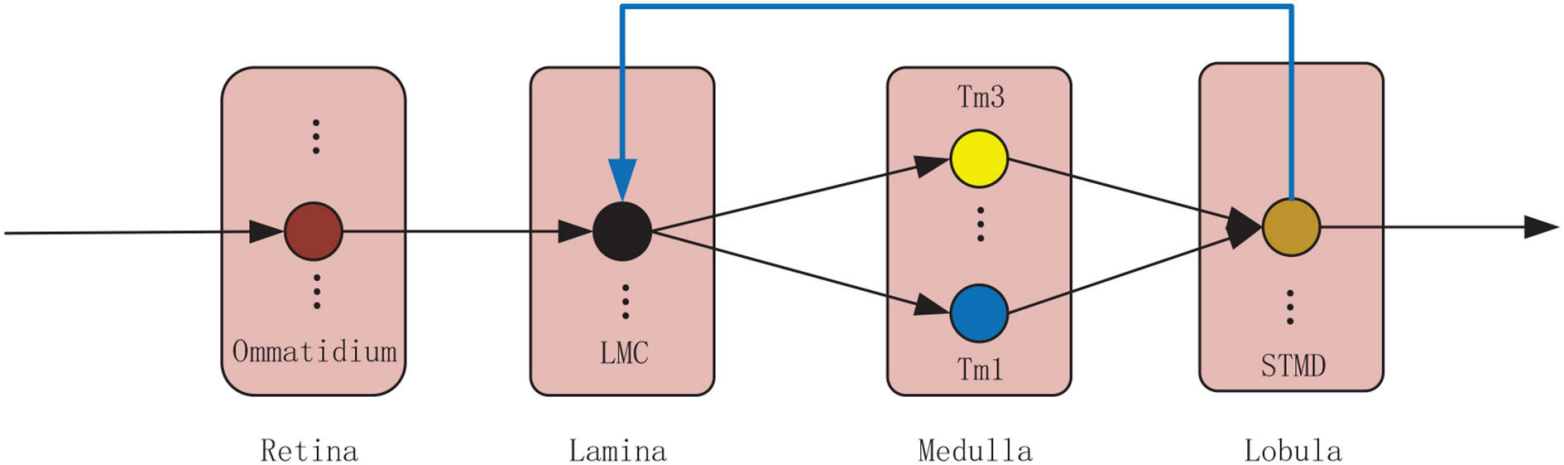
Wiring sketches of the proposed model. The proposed model consists of four neural layers, including retina, lamina, medulla and lobula. Each neural layer contains numerous neurons illustrated by colored circular node. The small target motion detectors (STMDs) relay their outputs to lamina neurons *via* a feedback mechanism.

The proposed model is composed of four neural layers and a feedback pathway (see Figure 1). The four neural layers are retina, lamina, medulla, and lobula. The four neural layers contain a number of specialized visual neurons coordinated together to detect small target motion. Specifically, visual information is captured by the ommatidia. The large monopolar cells (LMCs) receive the ommatidia output and feedback signal to calculate the change of brightness over time. The outputs of LMCs are further processed by Tm1 and Tm3 neurons in parallel. Finally, STMDs integrate the outputs of Tm1 and Tm3 neurons to detect the motion of small targets. In the following, the proposed model will be introduced in detail.

### 3.1. Retina

**Figure 2:**
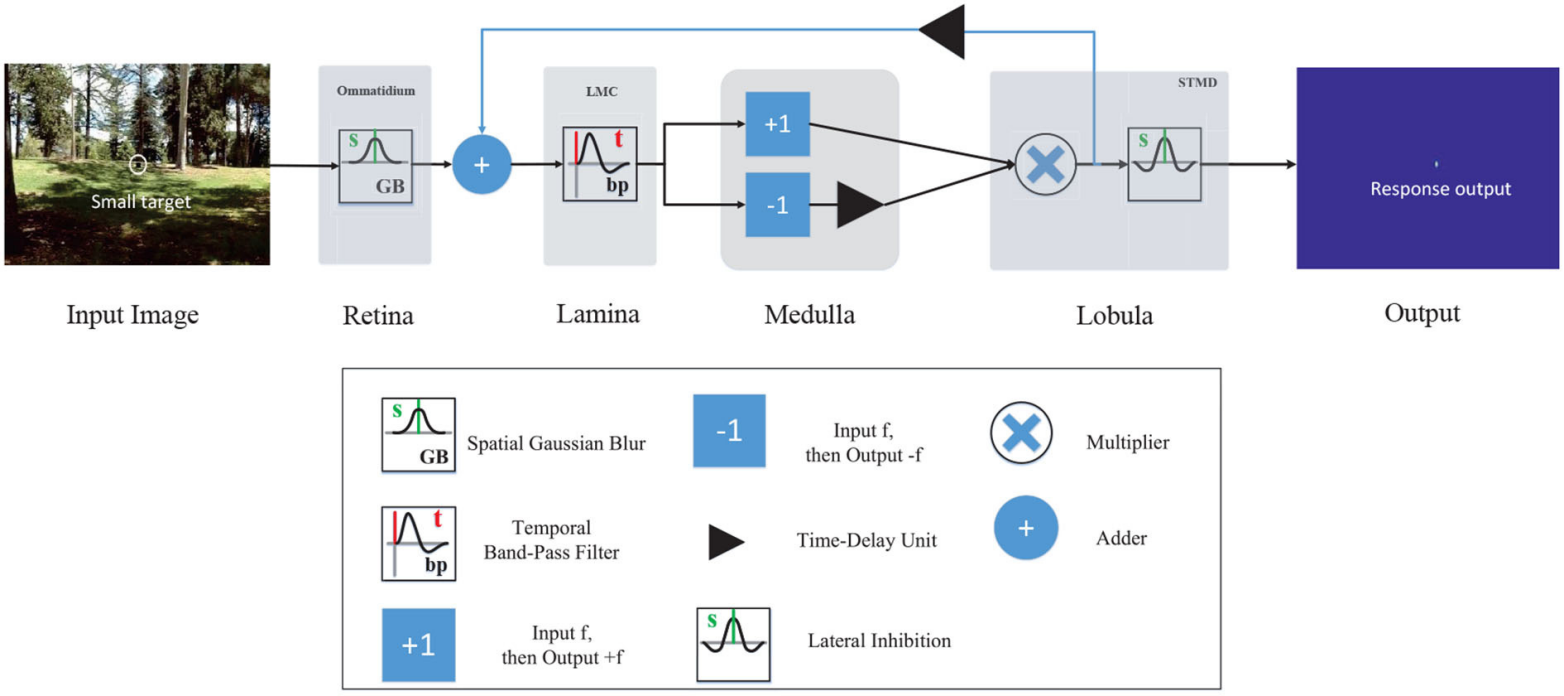
Schematic illustration of the feedback STMD (FSTMD) model. Visual information perceived by the retina layer is further processed in several layers of neuropil including the lamina layer, medulla layer, and lobula layer. The feedback signal is propagated to the lamina layer *via* feedback pathway to mediate neural responses.

The retinal layer contains a number of ommatidia (Sanes and Zipursky, 2010; Borst and Helmstaedter, 2015), as shown in Figure 1. Each ommatidium receives visual stimuli from a small region of visual field (Warrant, 2017; Meglič et al., 2019). In FSTMD, the ommatidium is modeled by a spatial Gaussian filter (see Figure 2) to smooth the luminance signal of each pixel. To be more precise, let *I*(*x, y, t*) ∈ *R* denote the brightness value captured by each ommatidium, where *x*, *y*, and *t* are spatial and temporal field positions. Then, the output of an ommatidium *P*(*x, y, t*) is described by


(1) $$\begin{array}{l} P\left(x,y,t\right)=\left[I\left(\cdot ,\cdot ,t\right)*G_{\sigma_{1}}\right]\left(x,y\right), \end{array}$$

where * denotes convolution; *G*_σ_1__(*x, y*) is a Gaussian function, defined as


(2) $$\begin{array}{l} G_{\sigma_{1}}\left(x,y\right)=\frac{1}{2\pi \sigma_{1}^{2}}e^{\frac{-\left(x^{2}+y^{2}\right)}{2\sigma_{1}^{2}}}, \end{array}$$

where σ_1_ is the standard deviation of the Gaussian function.

### 3.2. Feedback pathway

As shown in Figure 2, the LMC input signal is defined by adding the time-delay feedback signal with the ommatidium output, that is


(3) $$\begin{array}{l} P_{TF}\left(x,y,t\right)=P\left(x,y,t\right)+D_{F}\left(x,y,t\right), \end{array}$$

where *D*_*F*_(*x, y, t*) denotes the time-delay feedback signal. It is defined by convolving the STMD output *D*(*x, y, t*) with a Gamma kernel Γ_*n*_4_, τ_4__(*t*), that is


(4) $$\begin{array}{l} D_{F}\left(x,y,t\right)=a\left(D\left(x,y,\cdot \right)*\Gamma_{n_{4},\tau_{4}}\right)\left(t\right), \end{array}$$

(5) $$\begin{array}{l} \Gamma_{n,\tau }\left(t\right)={\left(nt\right)}^{n}\frac{e^{\frac{-nt}{\tau }}}{\left(n-1\right)!\cdot \tau^{n+1}}, \end{array}$$

where *a* (0 < |*a*| < 1) is the feedback constant; *n*_4_ and τ_4_ are the order and time constants of the Gamma kernel (De Vries and Príncipe, 1991), respectively.

### 3.3. Lamina

As shown in Figure 1, the lamina layer consists of large monopolar cells (LMCs) (Borst, 2009), which are postsynaptic neurons of the ommatidia and are sensitive to changes in brightness (Tuthill et al., 2013; Clark and Demb, 2016). They show strong responses to increase and decrease of brightness (Freifeld et al., 2013; Behnia et al., 2014; Li et al., 2017). In the proposed vision model, LMC is simulated by a time domain band-pass filter to extract brightness changes from the input signal. Mathematically, the LMC output *L*(*x, y, t*) is calculated by convolving the output of ommatidium *P*_*TF*_(*x, y, t*) with a kernel *H*(*t*), that is


(6) $$\begin{array}{l} L\left(x,y,t\right)=\left(P_{TF}\left(x,y,\cdot \right)*H\right)\left(t\right), \end{array}$$

where *H*(*t*) is defined by


(7) $$\begin{array}{l} H\left(t\right)=\Gamma_{n_{1},\tau_{1}}\left(t\right)-\Gamma_{n_{2},\tau_{2}}\left(t\right), \end{array}$$

where *n* and τ denote the order and time constants of the Gamma kernel.

### 3.4. Medulla

The medulla neurons, including Tm1 and Tm3 (Takemura et al., 2013; Fu et al., 2019), as illustrated in Figure 1. Tm3 neuron responds to brightness increase (Joesch et al., 2010; Clark et al., 2011); on the contrary, the Tm1 neuron responds to the decrease of brightness, and the response of the Tm1 neuron is relative to the Tm3 neuron with time delay at the same spatial positions. In the FSTMD, Tm1 and Tm3 neurons are simulated by half-wave rectifiers. The output of Tm3 neuron *S*_*ON*_(*x, y, t*) is defined by the positive part of the output of LMC *L*(*x, y, t*), that is


(8) $$\begin{array}{l} S_{ON}\left(x,y,t\right)=[L\left(x,y,t\right){]}^{+}. \end{array}$$

Meanwhile, the output of Tm1 neuron *S*_*OFF*_(*x, y, t*) is defined by convolving the negative part of the output of LMC *L*(*x, y, t*) with a kernel Γ_*n*_3_, τ_3__(*t*), that is


(9) $$\begin{array}{l} S_{OFF}\left(x,y,t\right)=\left({\left[L\left(x,y,\cdot \right)\right]}^{-}*\Gamma_{n_{3},\tau_{3}}\right)\left(t\right). \end{array}$$

### 3.5. Lobula

As can be seen from Figure 1, the lobula layer contains plenty of STMDs which integrate the signal from medulla neurons including Tm3 and Tm1 (Geurten et al., 2007; Clark et al., 2011). In the proposed visual system, the output of STMD neuron is defined by multiplying the output of Tm3 neuron *S*_*ON*_(*x, y, t*) with the output of Tm1 neuron *S*_*OFF*_(*x, y, t*) to detect small target motion, that is


(10) $$\begin{array}{l} D\left(x,y,t\right)=S_{ON}\left(x,y,t\right)\times S_{OFF}\left(x,y,t\right). \end{array}$$

**Figure 3:**
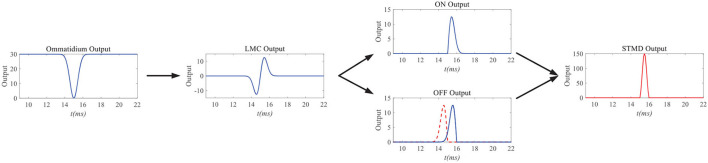
Neuron responds of the STMD pathway. When a small black target passes through a pixel point (*x, y*), the ommatidium output first drops to zero and then rises to its original position. The ommatidium output transmits to large monopolar cell (LMC) to calulate the changes of brightness over time *t*. LMC output further conveys to medulla neurons to perform parallel processing. Finally, STMD neuron integrate Tm3 neuron response and Tm1 neuron response to detect small target at pixel point (*x, y*).

Figure 3 simulates the neuron responses to the STMD pathway. When a small black target passes through a pixel point (*x, y*), the ommatidium output first drops to zero and then rises to its original position. It is worth noting that the drop and rise of ommatidium output are caused by the arrival and departure of the small target at pixel point (*x, y*). The ommatidium output transmits to LMC to calculate the changes of brightness over time *t*. LMC output further conveys to medulla neurons to perform parallel processing. Tm3 neuron responds to brightness increase; on the contrary, the Tm1 neuron responds to the decrease in brightness, and the response of the Tm1 neuron is relative to the Tm3 neuron with time delay at the same spatial positions. The time-delay length is defined as the ratio of the small target width to its velocity *v*. Finally, the STMD neuron integrates the Tm3 neuron response and Tm1 neuron response to detect a small target at pixel point (*x, y*).

**Figure 4:**
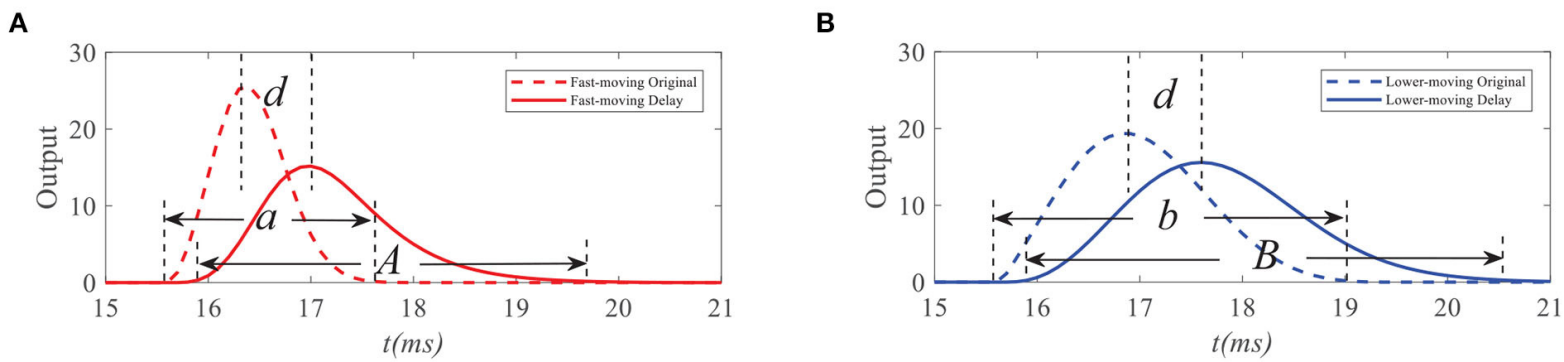
Original STMD outputs and feedback delay signal to small targets with different velocities. *a, A* (or *b, B*) denote the response durations of STMD output and feedback delay signal to fast-moving (or slow-moving) target, respectively **(A,B)**. *d* denotes the time-delay length.

**Figure 5:**
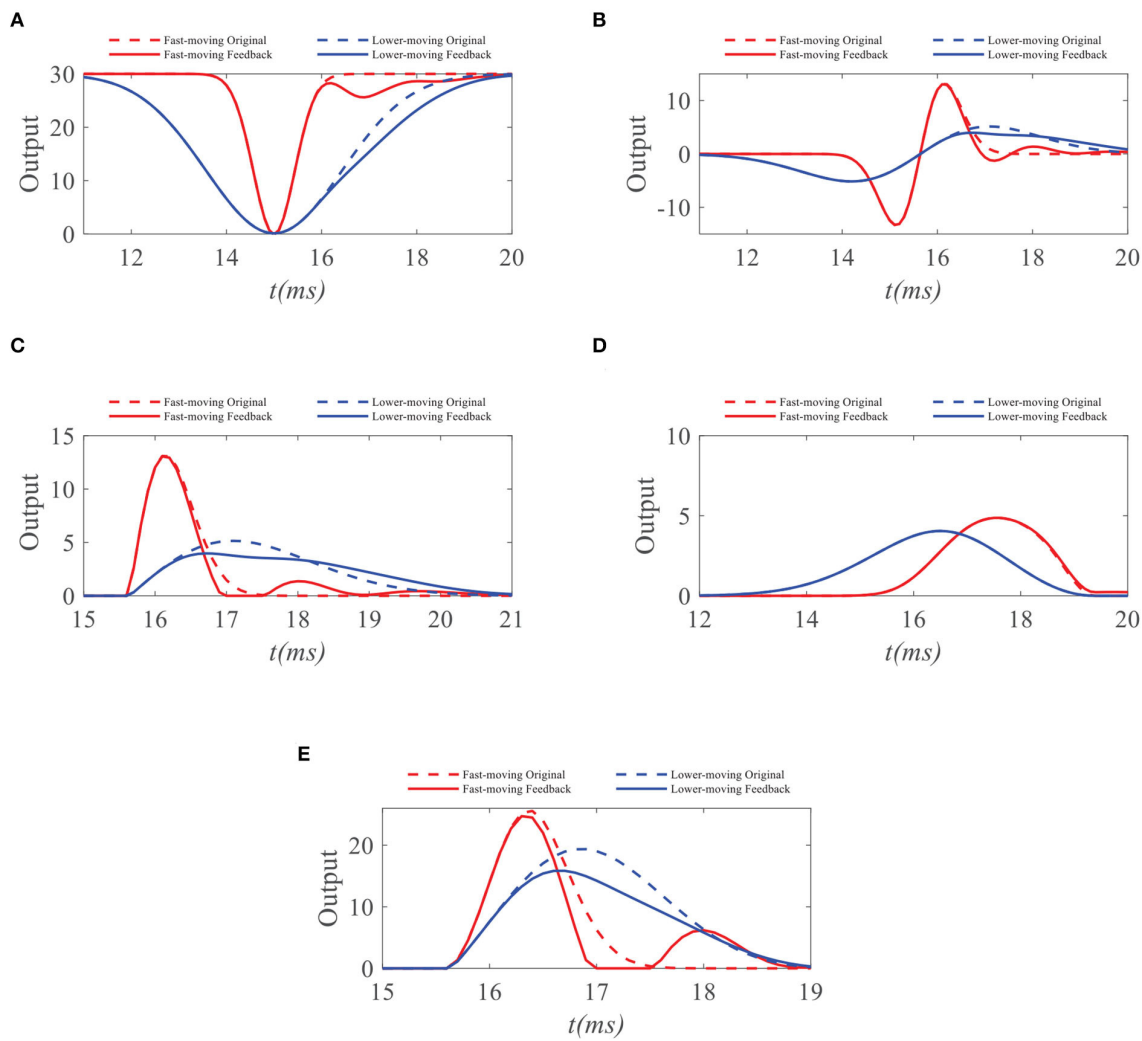
**(A)** Original ommatidia outputs and feedback ommatidia outputs to small targets with different velocities. The feedback signal weakens the ommatidium output to the slow-moving target in the second half of its response duration. In contrast, the ommatidium output to the fast-moving target receives minor inhibition from the feedback signal. Meanwhile, feedback signals have no effect on small targets with different velocities in the first half of their response durations, since STMD neuron has not yet responded when the small target enters the pixel point. **(B)** Original LMC outputs and feedback LMC outputs to small targets with different target velocities. The peak value of LMC output for fast-moving target maintains unchanged and the peak value of LMC output for slow-moving target has declines. **(C)** The original ON outputs and feedback ON outputs to small targets with different target velocities. The peak value of ON output to lower-moving target reduces, but the peak values of ON outputs to fast-moving target hardly change. **(D)** The original OFF outputs and feedback OFF outputs to small targets with different target velocities. **(E)** The peak values of OFF outputs to target with different velocities hardly change. The original STMD outputs and feedback STMD outputs to small targets with different target velocities. The STMD output reduction with a fast-moving target is significantly lower than a lower-moving target. Notice that there is a small perturbation in the STMD output of a fast-moving target. However, the peak value of small perturbation is lower than the maximum value of STMD output. Therefore, the small perturbation will be filtered by the region maximization operation.

To demonstrate the working mechanism of feedback, we first analyze the STMD outputs and feedback signals to the small target with different velocities. We simulate response outputs of four neural layers and feedback signals to small targets with different velocities, as shown in Figures 4, 5. Figures 4A,B shows the response durations of the STMD and feedback signals to small targets with different velocities. The response duration of STMD is defined by the time of the object completely covering a pixel point. When the target velocity is *v*, the response duration of STMD is controlled by 1/*v*. A longer response duration means it takes longer for the object to cover the pixel, which means the target velocity is slower. We express this relationship as *a* = *f*(1/*v*), where *f* is an increasing function. The feedabck signal is a time-delayed form of STMD output, where the response duration and time-delay length are controlled by the parameters *n*_4_ and τ_4_, respectively. Notice that the response duration of the feedback signal is larger than that of the STMD output (i.e., *A* > *a*) because of the convolutional operation.

We further compare the neural outputs of each neural layer to small targets with different velocities after negative feedback. As can be seen from Figure 5A, the feedback signal weakens the ommatidium output to a slow-moving target in the second half of its response duration. In contrast, the ommatidium output to the fast-moving target does not receive any inhibition from the feedback signal in the second half of its response duration, although a little disturbance appears outside the response duration. Meanwhile, feedback signals have no effect on small targets with different velocities in the first half of their response durations. The reason for this is that the STMD neuron has not yet responded when the small target enters the pixel point. In Figure 5B, we provide the LMC outputs to small targets with different velocities. We can see that the peak value of LMC output to slow-moving target has a decline, but maintains unchanged to fast-moving target. Figures 5C,D displays the medulla neurons the outputs to small targets with different velocities. Since the ON outputs are defined by the positive part of LMC outputs, the maximum value of ON output to a slow-moving target is suppressed by the feedback signal. In comparison, the maximum values of OFF outputs with and without feedback remain identical, because the feedback signal has a minor effect on the negative part of LMC outputs. Finally, we compare the STMD neural outputs with and without feedback in Figure 5E. As can be seen, the maximum value of the STMD output for a slow-moving target significantly decreases after feedback, whereas that of the STMD output for a fast-moving target hardly changes. Although there is a small perturbation in the STMD output to fast-moving target, the peak value of the perturbation is lower than the maximum value of STMD output and can be filtered out by the max-pool operation.

Comparing the STMD outputs to small targets with different velocities, we can find that if the time-delay length *d* is larger than half of the response duration of the feedback signal (i.e., *d* > *A*/2), the STMD output will maintain their maxima after subtracting the feedback signal. Thus, we obtain


(11) $$\begin{array}{l} d>\frac{A}{2}>\frac{a}{2}=\frac{f\left(1/v\right)}{2}, \end{array}$$

(12) $$\begin{array}{l} v>\frac{1}{f^{-1}\left(2d\right)}, \end{array}$$

It means that when the time-delay length *d* is fixed, the feedback signal has a small effect to targets with velocity *v* > 1/*f*^−1^(2*d*); meanwhile, if velocity *v* < 1/*f*^−1^(2*d*), the STMD output will be significantly reduced by feedback signal.

As shown in Figure 2, FSTMD implements a lateral inhibition mechanism on *D*(*x, y, t*) for size selectivity, that is


(13) $$\begin{array}{l} D_{w}\left(x,y,t\right)={\left[\left(D\left(\cdot ,\cdot ,t\right)*W\right)\left(x,y\right)\right]}^{+}, \end{array}$$

(14) $$\begin{array}{l} W\left(x,y\right)=A{\left[g\left(x,y\right)\right]}^{+}+B{\left[g\left(x,y\right)\right]}^{-}, \end{array}$$

(15) $$\begin{array}{l} g\left(x,y\right)=G_{\sigma_{2}}\left(x,y\right)-eG_{\sigma_{3}}\left(x,y\right)-\rho , \end{array}$$

where *W*(*x, y*) represents the lateral inhibition kernel; [*x*]^+^ and [*x*]^−^ denote max(*x*, 0) and min(*x*, 0), respectively; *A, B*, *e*, ρ are constants.

Finally, we give a detection threshold θ and compare it with the model output *D*_*w*_(*x, y, t*). If the output *D*_*w*_(*x, y, t*) is greater than the threshold θ, then we believe that a small target is detected at position (*x, y*) and time *t*.

## 4. Existence and uniqueness of solutions and algorithm

We propose the time-delay feedback vision system for small target motion detection. It designs a feedback pathway by transmitting information from output-layer neurons to lower-layer interneurons, forming a mathematical nonlinear dynamical system in infinite dimensional space. In order to estimate the range of the feedback constant *a* and propose the convergent algorithm, we need to prove the existence and uniqueness of the solutions to the nonlinear dynamical system. In the following, the existence and uniqueness of solutions to the nonlinear dynamic system will be analyzed by applying Schauder's fixed point theorem and contraction mapping theorem. We first introduce some basic facts to be used in this paper.

Let *L*^*p*^(Ω) be the space of real valued measurable functions *u* such that |*u*(*t*)|^*p*^ is Lebesgue integrable and the corresponding norm is given by


$$\begin{array}{l} ||u{||}_{L^{p}\left(\Omega \right)}=\{\begin{array}{l} {\left(\underset{\Omega }{\int }|u\left(t\right){|}^{p}dt\right)}^{\frac{1}{p}},\text{ for }1\leq p<\infty ,\\ ess\underset{t\in \Omega }{\sup}|u\left(t\right)|,\text{ for }p=\infty . \end{array} \end{array}$$

** Definition 1**. (Completely Continuous Operator Li et al., 2006) Let *X* be a Banach space and let *T* : *X* → *X* be an operator. *T* is said to be compact if it maps bounded sets of *X* into a sequentially compact set. Moreover, *T* is said to be completely continuous, if it is continuous and compact.

**Theorem 1**. (Kolmogorov-Riesz Hanche-Olsen and Holden, 2010) A subset *F* of $L^{2}\left(\left[0,T\right],R_{+}\right)$ is a sequentially compact set if, and only if,

(i) *F* is bounded,

(ii) for every ϵ > 0 there is some δ > 0 so that, for every *f* ∈ *F* and *h* with 0 < *h* < δ,


(16) $$\begin{array}{l} \int_{\left[0,T\right]}|f\left(t+h\right)-f\left(t\right){|}^{2}dt<\epsilon . \end{array}$$

Next, we recall two fixed point theorems, which help us analyze the existence and uniqueness of the solutions to the equation.

**Theorem 2**. (Banach Fixed Point Theorem Sousa et al., 2019) Let *X* be a Banach space and *T* : *X* → *X* is a contraction mapping. Then, *T* has a unique fixed point in *X*.

**Theorem 3**. (Schauder's Point Theorem Khan et al., 2011) Let *K* be a closed convex subset of a Banach space *X*. If *T* : *K* → *K* is continuous and *T*(*K*) is relatively compact, then *T* has a fixed point in *K*.

The feedback system can be formulated as *D*(*x, y, t*) = *F*_3_(*F*_2_(*F*_1_(*P*(*x, y, t*)+*D*(*x, y, t*)))) = *F*(*P*(*x, y, t*)+*D*(*x, y, t*)), where *P*(*x, y, t*) is the retina output signal, *D*(*x, y, t*) is the output signal, and *F* denotes the composition of three functions *F*_1_, *F*_2_, and *F*_3_, where *F*_1_, *F*_2_, and *F*_3_ represent the system response functions of three neural layers lamina, medulla, and lobula, respectively. For the sake of convenience, we define the operator *F* on $L^{2}\left(R^{2}\times \left[0,T\right],R_{+}\right)$ by


(17) $$\begin{array}{ll} (FD)(x,y,t)= & \left[(I*G(x,y)*H)(t)+a(D(x,y,t\right)\\ & *\Gamma_{4}*H)(t){]}^{+}\times ([(I*G(x,y)*H\\ & +aD(x,y,t)*\Gamma_{4}*H{]}^{-}*\Gamma_{3}(t)), \end{array}$$

where


(18) $$\begin{array}{l} \Gamma_{n,\tau }\left(t\right)=\{\begin{array}{l} {\left(nt\right)}^{n}\frac{e^{\frac{-nt}{\tau }}}{\left(n-1\right)!\cdot \tau^{n+1}},\text{ for }t\geq 0,\\ 0,\text{ for }t<0. \end{array} \end{array}$$

Using $f^{+}=\frac{\left(|f|+f\right)}{2}$ and $f^{-}=\frac{\left(|f|-f\right)}{2}$, the Equation (17) is equivalent to


(19) $$\begin{array}{l} \left(FD\right)\left(t\right)=([\left(P*H\right)\left(t\right)+a\left(D*V\right)\left(t\right){]}^{-}*\Gamma_{3}\left(t\right))\\ \;\times [\left(P*H\right)\left(t\right)+a\left(D*V\right)\left(t\right){]}^{+}\\ \;=\frac{1}{2}(\left(|\left(P*H\right)\left(t\right)+a\left(D*V\right)\left(t\right)|*\Gamma_{3}\right)\left(t\right)\\ \;-\left[\left(P*S\right)\left(t\right)+a\left(D*K\right)\left(t\right)\right])\\ \;\times \frac{1}{2}(|\left(P*H\right)\left(t\right)+a\left(D*V\right)\left(t\right)|\\ \;+\left(P*H\right)\left(t\right)+a\left(D*V\right)\left(t\right)), \end{array}$$

where *H*(*t*) = Γ_1_(*t*) − Γ_2_(*t*), *V*(*t*) = (Γ_4_ * *H*)(*t*), *K*(*t*) = (Γ_3_ * *V*)(*t*), *S*(*t*) = (Γ_3_ * *H*)(*t*), *P*(*x, y, t*) = (*I*(·, ·, *t*) * *G*)(*x, y*) and Γ(*t*), *H*(*t*), *V*(*t*), *K*(*t*), *S*(*t*) ∈ *L*^*p*^(*R*)(1 ≤ *p* ≤ ∞).

Before investigating the main result in this paper, we list essential conditions:

(*H*_1_): *P*(*t*) ∈ *L*^2^([0, *T*]).

Based on (*H*_1_), we can derive the following theorem. Theorem 4 illustrates that the operator *F* defined on *L*^2^ is well-defined.

**Theorem 4**. Suppose that (*H*_1_) holds, then $F\left(L^{2}\left(\left[0,T\right],R_{+}\right)\right)\subset L^{2}\left(\left[0,T\right],R_{+}\right)$.

Proof. See the **Proof of Theorem 4** in the Appendix.

The following theorem illustrates that operator *F* is continuous, which is very important for the proof of existence and uniqueness of the solution to the equation.

**Theorem 5**. Assume that (*H*_1_) holds, then operator $F:L^{2}\left(\left[0,T\right],R_{+}\right)$ → $L^{2}\left(\left[0,T\right],R_{+}\right)$ is continuous.

Proof. See the **Proof of Theorem 5** in the Appendix.

From the previous discussion, it is clear that the operator *F* is continuous. To get the existence of the solution to the equation, we also need to illustrate that the operator *F* is a compact operator.

**Theorem 6**. Suppose that (*H*_1_) holds, then $F:L^{2}\left(\left[0,T\right],R_{+}\right)\to L^{2}\left(\left[0,T\right],R_{+}\right)$ is a compact operator.

Proof. See the **Proof of Theorem 6** in the Appendix.

According to Theorem 5 and Theorem 6, we immediately obtain that the operator *F* is a completely continuous operator. In the following theorems, the existence and uniqueness of solution are verified by applying Schauder's fixed point theorem and contraction mapping theorem. We first present the existence of solutions.

**Theorem 7**. Suppose that (*H*_1_) holds and *a* < 0, then the following results hold:

(i). If *Q*^2^ − 4*N*′φ = 0 and *aQ* + 1 > 0, then $a\in [-\frac{1}{2Q},0)$ and the Equation (19) has at least one solution on $B_{r}=\{D\in L^{2}\left(\left[0,T\right],R_{+}\right),||D{||}_{L^{2}}\leq r\}$, where


(20) $$\begin{array}{l} \;\frac{\left(aQ+1\right)-\sqrt{2Qa+1}}{2a^{2}N^{\prime }}\leq \\ r\leq \frac{\left(aQ+1\right)+\sqrt{2Qa+1}}{2a^{2}N^{\prime }}. \end{array}$$

(ii). If *Q*^2^ − 4*N*′φ > 0 and *aQ* + 1 > 0, then $a\in (-\frac{1}{Q},\frac{-1}{Q+2\sqrt{N^{\prime }\varphi }}]$ and the Equation (19) has at least one solution on $B_{r}=\{D\in L^{2}\left(\left[0,T\right],R_{+}\right),||D{||}_{L^{2}}\leq r\}$, where


(21) $$\begin{array}{l} \;\frac{\left(aQ+1\right)-\sqrt{{\left(aQ+1\right)}^{2}-4a^{2}N^{\prime }\varphi }}{2a^{2}N^{\prime }}\leq \\ r\leq \frac{\left(aQ+1\right)+\sqrt{{\left(aQ+1\right)}^{2}-4a^{2}N^{\prime }\varphi }}{2a^{2}N^{\prime }}. \end{array}$$

(iii). If *Q*^2^ − 4*N*′φ < 0 and *aQ* + 1 > 0, then $a\in [\frac{-1}{Q+2\sqrt{N^{\prime }\varphi }},0)$ and the Equation (19) has at least one solution $D\in B_{r}=\{D\in L^{2}\left(\left[0,T\right],R_{+}\right),||D{||}_{L^{2}}\leq r\}$, where


(22) $$\begin{array}{l} \;\frac{\left(aQ+1\right)-\sqrt{{\left(aQ+1\right)}^{2}-4a^{2}N^{\prime }\varphi }}{2a^{2}N^{\prime }}\leq \\ r\leq \frac{\left(aQ+1\right)+\sqrt{{\left(aQ+1\right)}^{2}-4a^{2}N^{\prime }\varphi }}{2a^{2}N^{\prime }}. \end{array}$$

Proof. See the **Proof of Theorem 7** in the Appendix.

The following theorem gives the unique results of the solution to the nonlinear Equation (19).

**Theorem 8**. Assume that (*H*_1_) holds, then the following results hold:

(i). If *Q*^2^ − 4*N*′φ = 0, $a\in [-\frac{1}{2Q},0)$ and $r=\frac{\left(aQ+1\right)-\sqrt{2Qa+1}}{2a^{2}N^{\prime }}$, then there is unique one solution to the Equation (19) on $L^{2}\left(\left[0,T\right],R_{+}\right)$.

(ii). If *Q*^2^ − 4*N*′φ < 0, $a\in \left[\frac{-1}{Q+2\sqrt{N^{\prime }\varphi }},0\right)$ and $r=\frac{\left(aQ+1\right)-\sqrt{{\left(aQ+1\right)}^{2}-4a^{2}N^{\prime }\varphi }}{2a^{2}N^{\prime }}$, then there is unique one solution to the Equation (19) on $L^{2}\left(\left[0,T\right],R_{+}\right)$.

Proof. See the **Proof of Theorem 8** in the Appendix.

**Figure 6:**
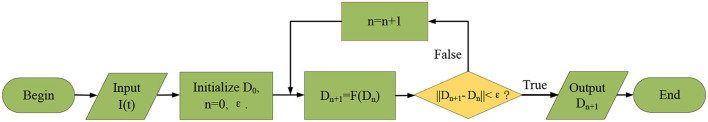
Schematic illustration of the algorithm flowchart.

In the following, we design a fixed point iteration algorithm to solve the nonlinear dynamic system (19), where the pseudocodes of our algorithm are listed in Algorithm 1, and the flowchart of the algorithm is shown in Figure 6. In the iterative process, when the error between *D*_*n*+1_(*x, y, t*) and *D*_*n*_(*x, y, t*) is lower than the preset error threshold 10^−3^, we stop the iteration and assume that *D*_*n*+1_(*x, y, t*) is model response output.

**Algorithm 1:**
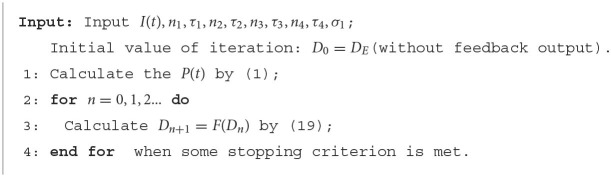
FSTMD Iterative algorithm.

**Feedback Constant Estimation:** From the theorem 8, we prove the nonlinear system has unique solution, which means the proposed algorithm to solve dynamic systems is stable. To determine the feedback constant *a*, we first estimate *Q*, *N*′, and φ by Theorem 7, and then substitute them into $a\in \left[\frac{-1}{Q+2\sqrt{N^{\prime }\varphi }},0\right)$ revealed in the condition (*ii*) of Theorem 8.

## 5. Experiment

**Figure 7:**
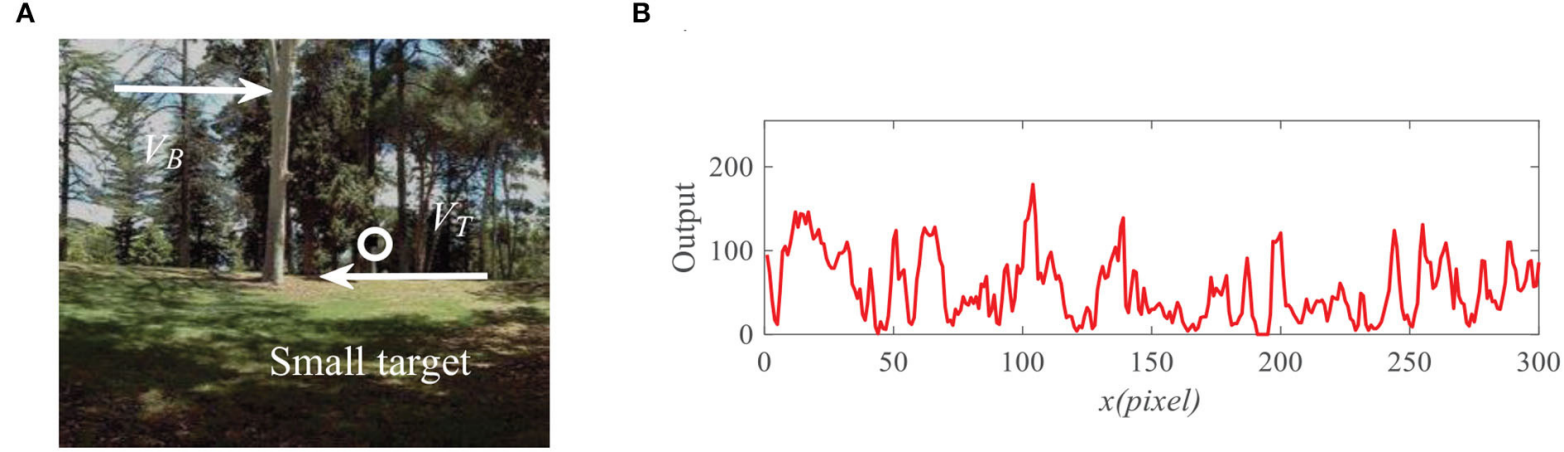
**(A)** Input image at time *t*_0_ = 430ms, where a small target (the black block) is moving against the cluttered natural background. Arrow *V*_*B*_ denotes motion direction of the background and *V*_*T*_ denotes motion direction of the small target. **(B)** Input signal with respect to *x* for the given *y*_0_ = 125 pixels and time *t*_0_ = 430ms.

In this section, we demonstrate the effectiveness of the proposed algorithm for detecting small target's motion against cluttered backgrounds. We evaluate the proposed FSTMD on the Vision Egg data set (Straw, 2008) and the RIST data set RIST Data Set^1^. The Vision Egg data set includes a number of synthetic image sequences, each of which displays a small target moving against a natural background image (see Figures 7A, 11A). Each synthetic video contains one or multiple small target motions, whose resolution and sampling frequency equate to 300 × 250 pixels and 1, 000 Hz, respectively. The RIST data set includes 16 videos, which were captured in real environments by using an action camera (GoPro Hero 6) at 240 fps. Each video contains an object that is moving in cluttered scenarios. All the experiments are performed under Windows 10 and MATLAB (R2017a) running on a computer with a Core i5 CPU at 3.10GHz with 16GB of memory. The parameters of the FSTMD model are shown in Table 1. The parameters of model consist of two parts, including the parameters of the four feed-forward neural layers and the feedback pathway. The parameters of the four feed-forward neural layers and their effect on the performance have been investigated and analyzed in the reference (Wang et al., 2018). In this paper, we choose the parameters to make the model satisfy the basic properties of STMD neurons such as size selectivity and velocity selectivity. The parameters of the feedback pathway *n*_4_ and τ_4_ can be tuned so that the appropriate optimal velocity and preferred velocity range of the model can be selected.

**Table 1:** Parameters of the feedback small target motion detectors (FSTMDs).

**Equations**	**Parameters**
(1)	σ_1_ = 1
(6)	*n*_1_ = 2, τ_1_ = 3, *n*_2_ = 6, τ_2_ = 9
(9)	*n*_3_ = 5, τ_3_ = 25
(4)	*n*_4_ = 5, τ_4_ = 10
(13)	σ_2_ = 1.5, σ_3_ = 3, *e* = 1, ρ = 0

**Figure 8:**
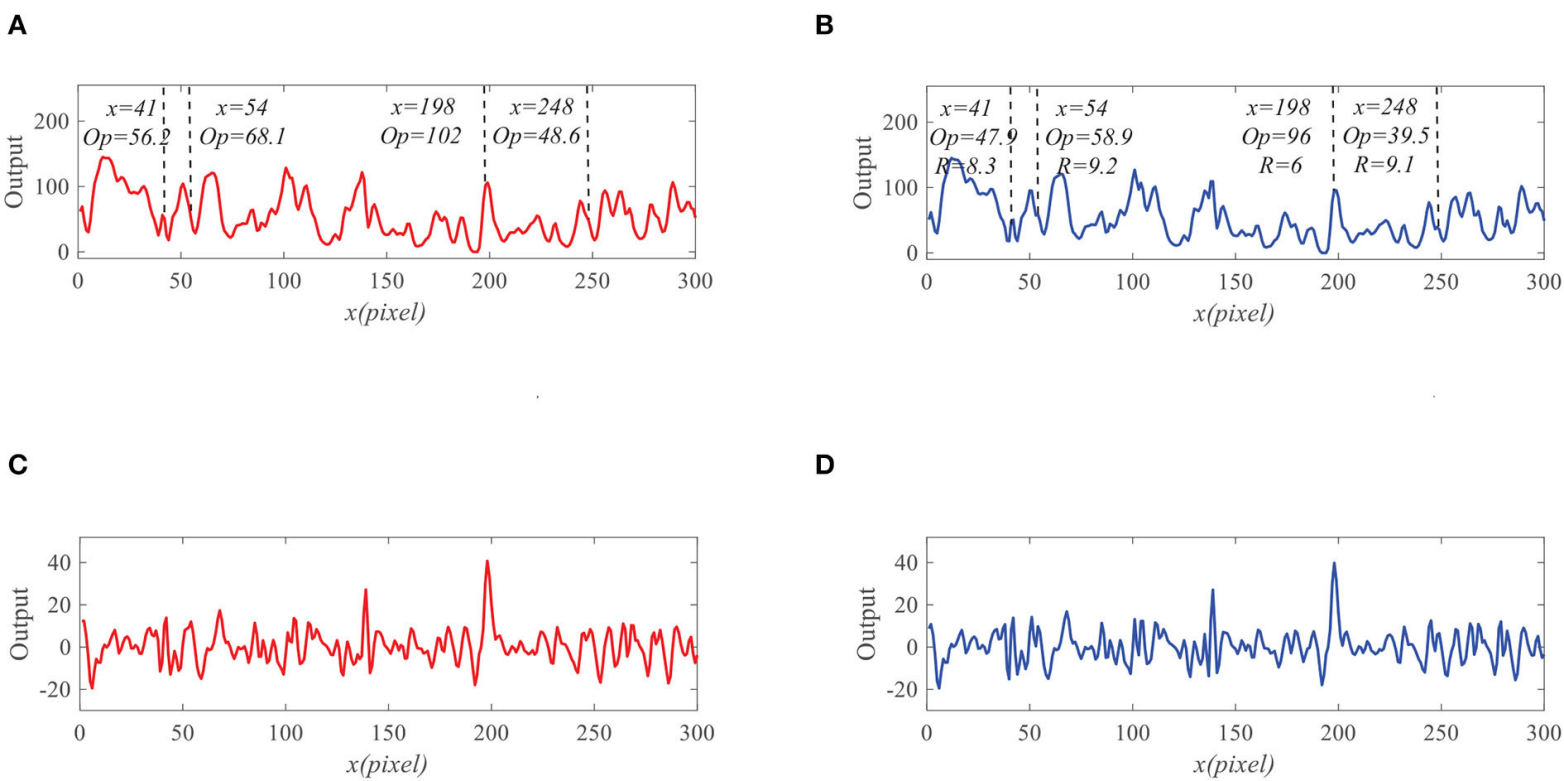
The outputs of the ommtidia and LMCs neurons. **(A)** The outputs of ommatidia without feedback. **(B)** The outputs of ommatidia with feedback. **(C)** The outputs of LMCs without feedback. **(D)** The outputs of LMCs with feedback.

To demonstrate the effectiveness of the proposed model, we present the neural outputs with and without feedback. Figure 7A shows the input image at time *t*_0_ = 430 ms, where a small black target (250 pixels/s) is moving against a cluttered background (150 pixels/s). We fix *y*_0_ = 125 pixels then display the input luminance signal *I*(*x, y*_0_, *t*_0_) with respect to *x* at time *t*_0_ in Figure 7B. It can be seen from Figure 7B that the input signal is quite complex and small target is submerged in background clutters. As shown in Figure 8A, the outputs of ommatidia are a smooth version of the input luminance signal *via* Gaussian blur. Figure 8B shows the feedback ommatidia results which are calculated by subtracting the feedback signal from the original ommatidia outputs. From Figure 8B, we can find that the ommatidia outputs with feedback decrease after experiencing negative feedback at *x* = 41, 54, 198, and 248. The outputs of LMCs in Figures 8C,D are given to show luminance change at time *t*_0_ at each pixel. In particular, a positive value of LMC output indicates luminance increases, while a negative value of LMC output represents a decrease in luminance at time *t*_0_.

**Figure 9:**
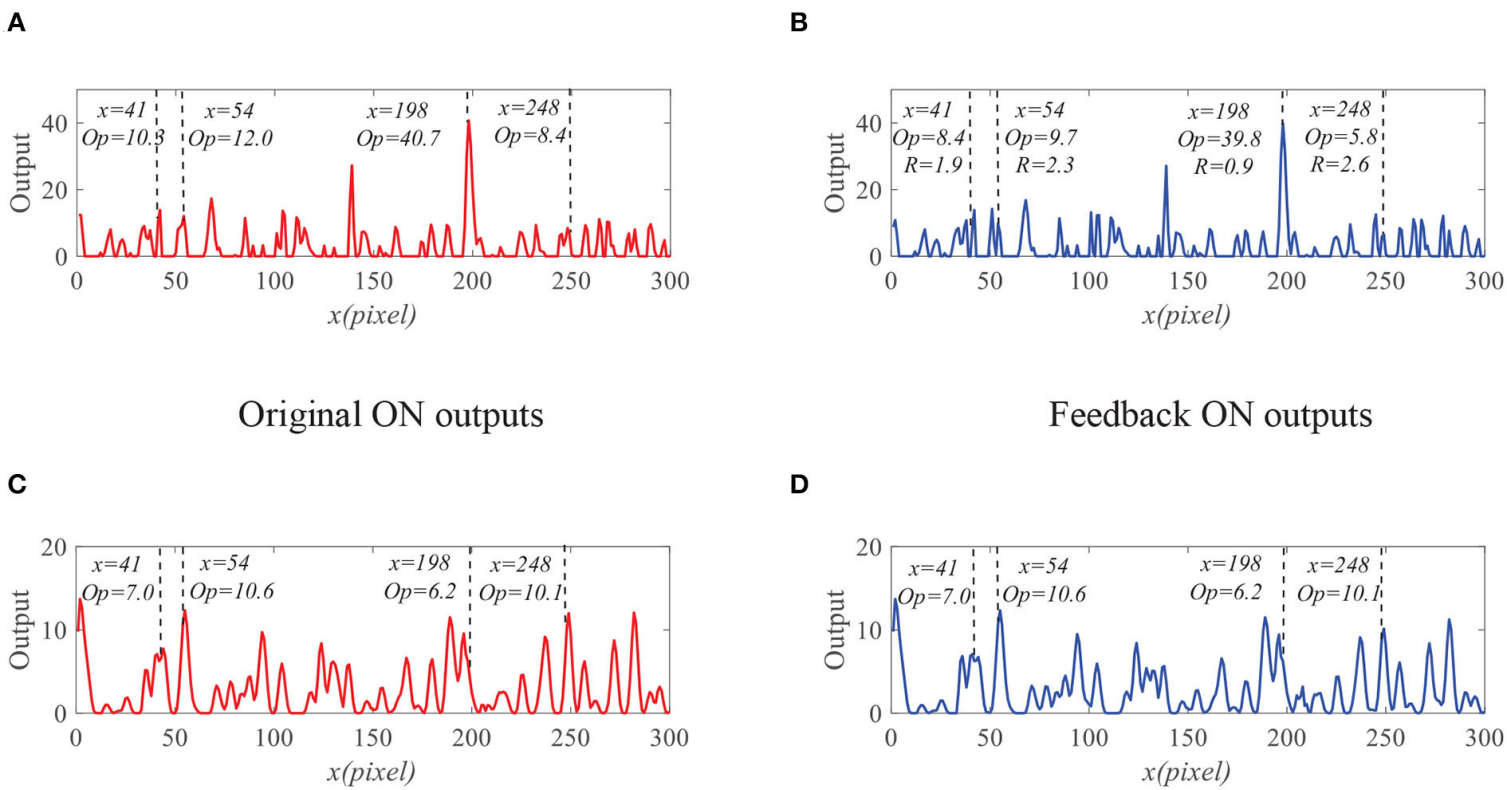
The outputs of the medulla neurons. **(A)** The outputs of ON without feedback. **(B)** The outputs of ON with feedback. **(C)** The outputs of OFF without feedback. **(D)** The outputs of OFF with feedback.

**Figure 10:**
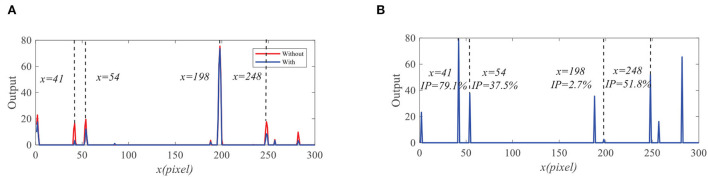
**(A)** The original STMDs outputs and feedback STMDs (FSTMDs) outputs. **(B)** The inhibition percentage (IP) of the different locations.

We further compare the outputs of the medulla neurons. Figure 9 displays the outputs of ON and OFF with and without feedback. We can see that the ON outputs decrease *via* feedback, while the OFF outputs hardly alter at *x* = 41, 54, 198, and 248. This is because that the feedback suppresses the positive part of LMC output to targets but has minor effect on the negative part of LMC outputs. In addition, the reduction of the ON output *R* = 0.9 at *x* = 198 is smaller than that of background false targets locations 1.9, 2.3, and 2.6. The outputs of STMDs are defined by multiplying the outputs of Tm3 neurons and Tm1 neurons and implementing a lateral inhibition. From Figure 10A, the outputs of STMDs with feedback are significantly lower than the original outputs of STMDs. In Figure 10B, we provide the inhibition percentage (IP) of small target positions and background false target locations. It can be seen from Figure 10B that the IP of the real target location is generally much smaller than that of background fake target locations. In particular, the IP of the fast-moving small target at position *x* = 198 is 2.7%, and the IPs of the slow-moving background false targets at position *x* = 41, 54, and 248 are 79.1, 37.5, and 51.8% respectively. The above results demonstrate that feedback significantly suppresses slow-moving background features.

To quantitatively compare with the existing STMD-based model, we define two metrics (Wang et al., 2018),

(23) $$\begin{array}{l} D_{R}=\frac{\text{number of true detections}}{\text{number of actual targets}}, \end{array}$$

(24) $$\begin{array}{l} F_{A}=\frac{\text{number of false detections}}{\text{number of images}}, \end{array}$$

where *D*_*R*_ denotes detection rate and *F*_*A*_ represents false alarm rate. If the pixel distance between the actual position of the target and the detection result is within a threshold (5 pixels), then we consider the detection result is correct.

**Figure 11:**
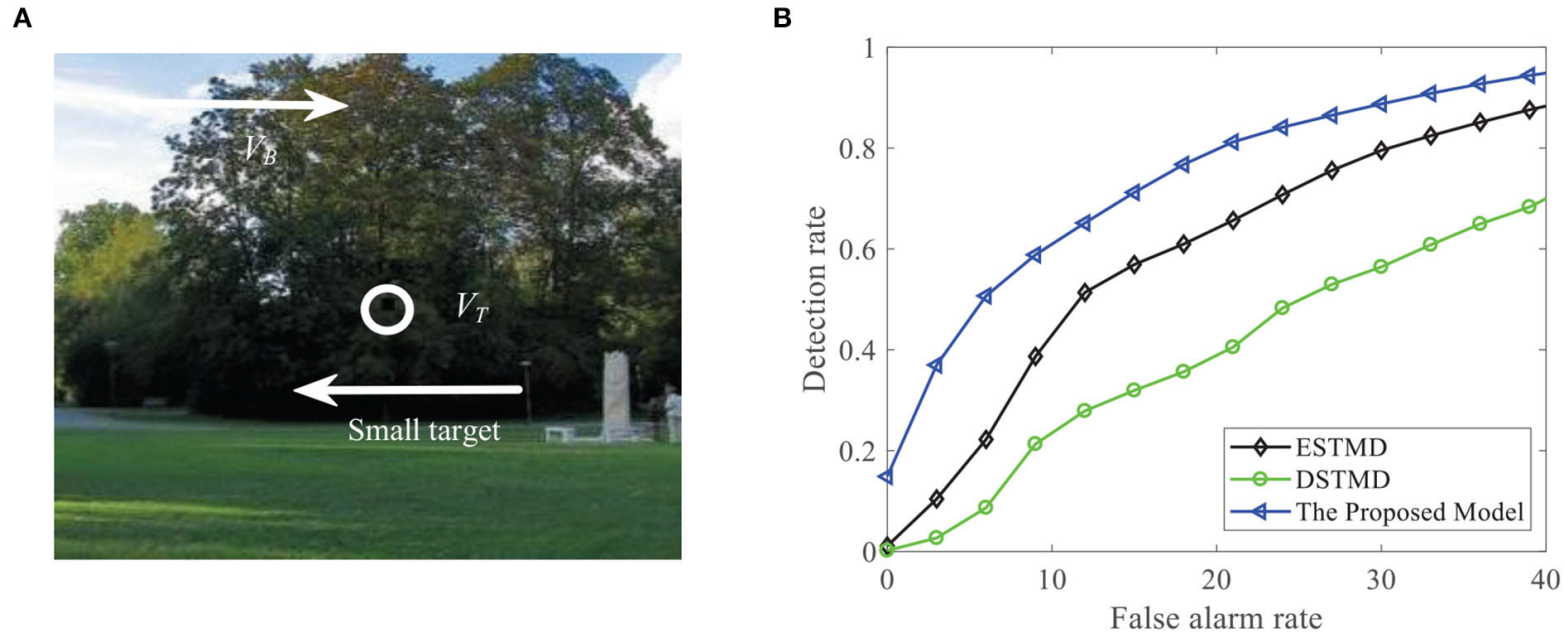
**(A)** A frame of the initial sequence. A small target is moving against the cluttered background. Arrow *V*_*B*_ denotes motion direction of the background and *V*_*T*_ denotes motion direction of the small target. **(B)** *D*_*R*_ − *F*_*A*_ curves of the four models for the initial image sequence.

**Figure 12:**
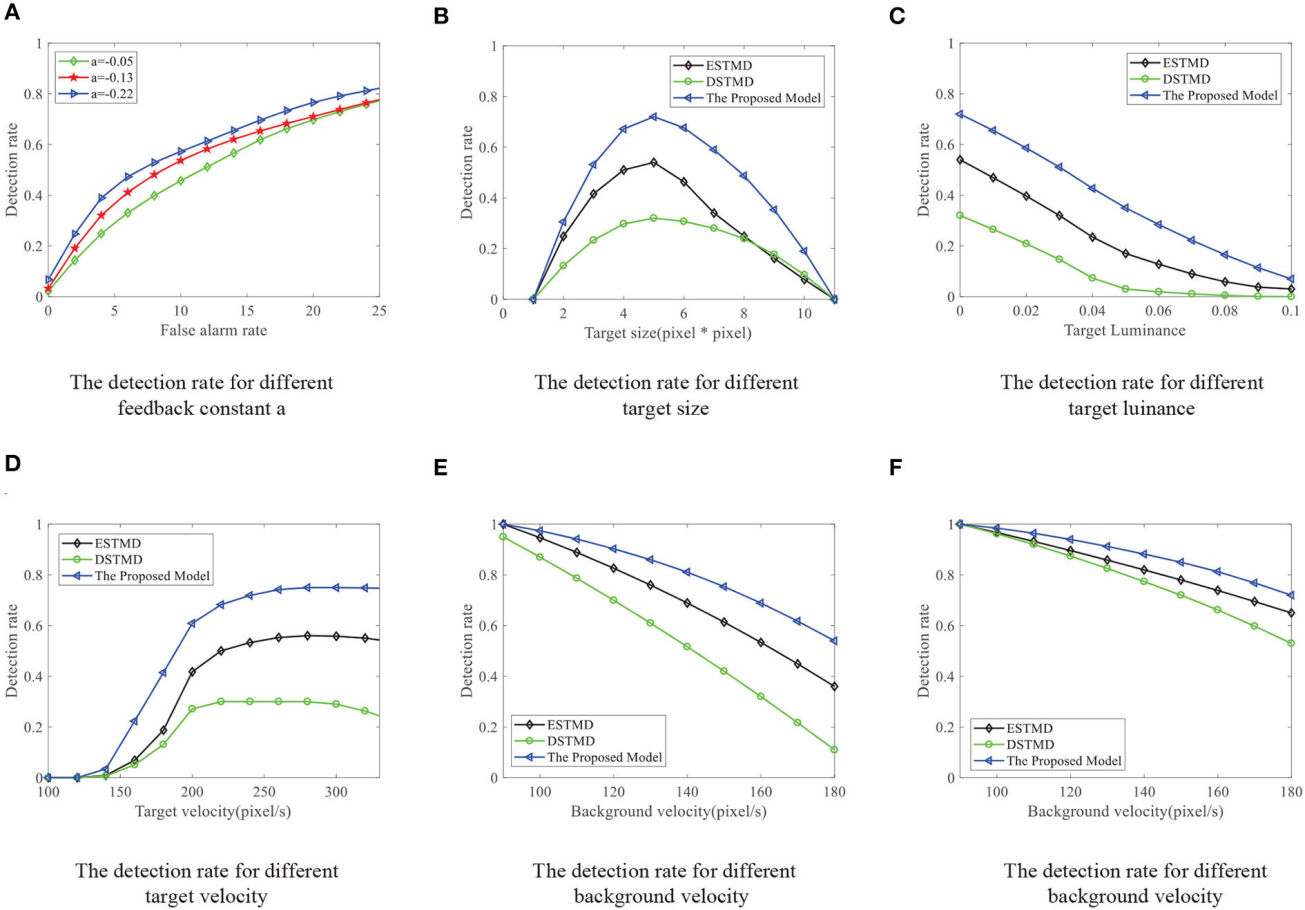
**(A)** The *D*_*R*_ − *F*_*A*_ curves of the FSTMD for different feedback constant *a*. **(B–F)** The *D*_*R*_ − *F*_*A*_ curves of the three models for the **(B)** different target sizes, **(C)** different target luminance, **(D)** different target velocities, **(E)** different background velocities, and **(F)** different background velocities (leftward motion) with the fixed false alarm rate (FA = 15).

We compare the performance of the proposed FSTMD with ESTMD (Wiederman et al., 2008) and DSTMD (Wang et al., 2018) models in terms of different target velocities, target sizes, target luminance, and background velocities. The details of the synthetic image sequences are listed in Table 2. Figure 11B shows the *D*_*R*_ − *F*_*A*_ curves of three models for the initial synthetic image sequence. It can be seen that the feedback model has higher detection rates compared with ESTMD and DSTMD for any false alarm rates. Figure 12A provides the detection result curves of the proposed model for different feedback constant *a*. These *D*_*R*_ − *F*_*A*_ curves demonstrate that with the decrease of feedback constant *a*, the detection performance of FSTMD model becomes better. The reason for this is that with the decrease of feedback constant *a*, the feedback signal enhances inhibition to slow-moving background false targets but has a small effect on fast-moving small targets. Figures 12B–F displays the detection rates of the three models concerning target size, target luminance, target velocity, and background velocity when the false alarm rate is equal to 15. The detection results of the three models for different target sizes are displayed in Figure 12B. It can be seen that the detection rates of three models show a significant decrease after reaching the highest point when the target size increases from 1 × 1 to 11 × 11. However, the feedback model achieves higher detection rates than other models with the increase in target size. In Figure 12C, we can observe that in the three models, all decrease with the increase in target luminance. It is also worth noting that the proposed model has much better detection performance than the ESTMD and DSTMD when the target luminance is less than 0.1. The results of detection for target velocity are presented in Figure 12D. It can be seen that the detection rates of DSTMD first go up and then slightly decline when the target velocity increases from 100 pixels/s to 330 pixels/s. The detection rates of feedback model and ESTMD increase with the increase of target velocity. However, the performance of feedback model in discriminating the motion of small target from the dynamic background has a superior advantage when target velocity exceed 200 pixels/s. The reason for this is that with the increase of target velocity, the suppression of feedback progressively weakens to a fast-moving target. Figures 12E,F show the results of the detection of the three models for different background velocities. Figures 12E,F show that the proposed model improves detection rates when the background velocity *v*_*B*_ is lower than 180 pixels/s. The reason is that when the background is moving slower than the target, the background false targets will receive stronger suppression by the feedback signal, which consequently leads to the detection rate of FSTMD that is higher than the baseline models.

**Table 2:** Details of the synthetic image sequences.

**Image parameter**	**Initial sequence**	**Test 1**	**Test 2**	**Test 3**	**Test 4**	**Test 5**
Target size (pixel × pixel)	5 × 5	1 × 1~11 × 11	5 × 5	5 × 5	5 × 5	5 × 5
Target luminance	0	0	0~0.1	0	0	0
Target velocity (pixel/s)	250	250	250	100~330	250	250
Background velocity (pixel/s)	150	150	150	150	50~180	50~180
Background motion direction	rightward	rightward	rightward	rightward	rightward	leftward
Background image	Figure 11A	Figure 11A	Figure 11A	Figure 11A	Figure 11A	Figure 11A

**Figure 13:**
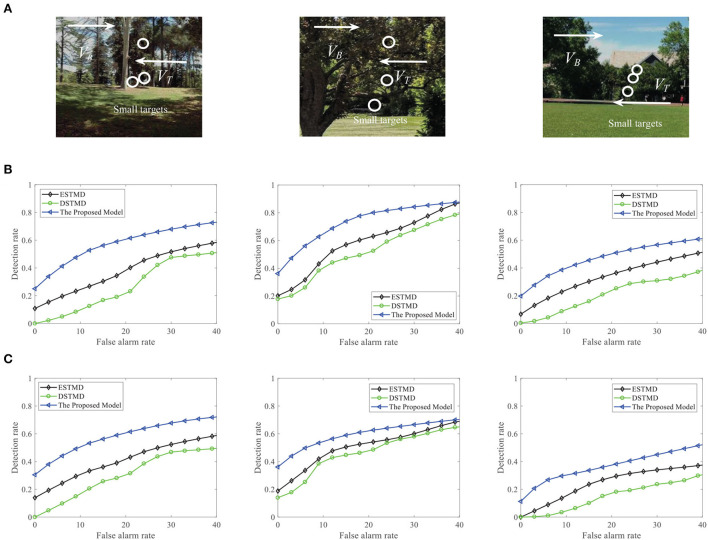
**(A)** A frame of the sequence. Small targets are moving against the different cluttered backgrounds. Arrow *V*_*B*_ denotes motion direction of the background and *V*_*T*_ denotes motion direction of the small target. **(B)** The *D*_*R*_ − *F*_*A*_ curves of the three models for different background with a target. **(C)** The *D*_*R*_ − *F*_*A*_ curves of the three models for different background with three targets.

**Figure 14:**
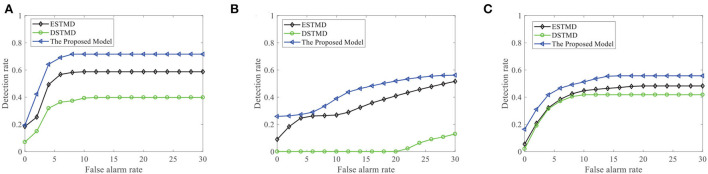
The detection result of the proposed FSTMD on three real videos in comparison to the elementary STMD (ESTMD) and directionally selective (DSTMD) models. **(A)** Real video 1 (GX010303), **(B)** Real video 2 (GX010335), and **(C)** Real video 3 (GX010241). The FSTMD achieves better performance than the other two models on all real videos.

We further demonstrate the performance of the FSTMD model by changing the background image (see Figure 13A). Figure 13B displays the *D*_*R*_ − *F*_*A*_ curves of the three models to detect the motion of a small target in different backgrounds. Meanwhile, the *D*_*R*_ − *F*_*A*_ curves of the three models to detect the motion of three small targets in different backgrounds are presented in Figure 13C. From Figures 13B,C, we can see that the FSTMD has a better performance than the existing models for different backgrounds and numbers of the target. In addition, we also evaluate the models on three real videos and display their detection result curves in Figure 14. The videos are randomly selected from the RIST data set, each of which displays a moving small target against the cluttered background. The corresponding video numbers are given in the caption. As can be seen, the FSTMD outperforms the ESTMD and DSTMD models in all three videos. Specifically, its detection rate is always higher than those of the other two models at any false alarm rate. Combined with the above experimental results, it shows that our algorithm is practical and feasible.

## 6. Conclusion

In this paper, we have proposed a time-delay feedback vision system for detecting a small moving target in cluttered backgrounds. Our model contains four neural layers and a time-delay feedback pathway. The four neural layers process motion information in a feed-forward manner, and the feedback pathway propagates the output of the model to lower-layer neurons for weakening slow-moving background false positive responses. In order to determine the feedback intensity, we prove the existence and uniqueness of the solution to the nonlinear dynamic system formed by the feedback loop and designed an iterative algorithm to solve the approximate solution of the model. To demonstrate the advantages of the proposed model, we apply the proposed model to detect small target motions in cluttered backgrounds. The experimental results show that the proposed model maintains a minor effect to fast-moving objects, while significantly suppressing those with lower velocities. Finally, we compare the performance of the proposed model with the existing models, experimental results show that the proposed model is able to improve detection performance for small targets with velocities higher than that of the complex background.

However, the performance of STMD is crucially dependent on the contrast between the background and target. Insects pursue prey based on the contrast between the background and target. The proposed model also uses the contrast between background and target to detect small targets. It conforms to the laws of biological vision. When the contrast between the background and target is small, the performance of the model is low. In the future, some contrast enhancement methods, such as histogram equalization and gray transformation, can be integrated into the model to overcome the problem of low contrast. In addition, this paper only analyzes the fixed time-delay feedback system, which is formed by transmitting the information from the output layer to the lamina layer in the STMD pathway. There may be some problems that have not been considered in the STMD vision system. For example, we may consider the time-varying feedback cases and other neural layers' feedback types in the STMD pathway.

## Data availability statement

The original contributions presented in the study are included in the article/Supplementary material, further inquiries can be directed to the corresponding authors.

## Author contributions

JL: writing, experiments, and theoretical analysis. HL and JP: ideas of the project and review. HW, MX, and HC: experiments and writing-review. All authors contributed to the article and approved the submitted version.

## Funding

This work was supported in part by the National Natural Science Foundation of China under Grant Nos. 12031003 and 11771347, in part by the China Postdoctoral Science Foundation under Grant No. 2021M700921.

## Conflict of interest

The authors declare that the research was conducted in the absence of any commercial or financial relationships that could be construed as a potential conflict of interest.

## Publisher's note

All claims expressed in this article are solely those of the authors and do not necessarily represent those of their affiliated organizations, or those of the publisher, the editors and the reviewers. Any product that may be evaluated in this article, or claim that may be made by its manufacturer, is not guaranteed or endorsed by the publisher.

## Footnotes

^1^https://sites.google.com/view/hongxinwang-personalsite/download/ (accessed on April 6, 2020).
