# Supplementary material for: Mathematical study of neural feedback roles in small target motion detection
Source: Front Neurorobot. 2022 Sep 20;16:984430. doi: 10.3389/fnbot.2022.984430 (PMC9530796; doi:10.3389/fnbot.2022.984430)
Supplement: Supplementary file 1 [file Presentation_1.pdf]

The Proof of Theorem

Jun Ling [1], **Hongxin Wang** [2,3], **Mingshuo Xu** [1], **Hao Chen** [1], **Haiyang Li** [1,*] **and Jigen Peng** [1,*]

[1] *School of Mathematics and Information Science, Guangzhou University, Guangzhou 510006, China*
[2] *Machine Life and Intelligence Research Center, Guangzhou University, Guangzhou 510006, China*
[3] *Computational Intelligence Lab (CIL), School of Computer Science, University of Lincoln, Lincoln, LN6 7TS, UK*

Correspondence*:
Haiyang Li & Jigen Peng
fplihaiyang@126.com & jgpeng@gzhu.edu.cn

In this section, we mainly prove all the Theorems. Let's rewrite the equation as follows

$$\begin{aligned}(FD)(t) =&([(P*H)(t)+a(D*V)(t)]^{-}*\Gamma_3(t))\\ &\times [(P*H)(t)+a(D*V)(t)]^{+}\\ =&\frac{1}{2}((|(P*H)(t)+a(D*V)(t)|*\Gamma_3)(t)\\ &-((P*S)(t)+a(D*K)(t)))\\ &\times\frac{1}{2}(|(P*H)(t)+a(D*V)(t)|\\ &+(P*H)(t)+a(D*V)(t)),\end{aligned} \tag{1}$$

Adopting Hölder's inequality and (H_1), we deduce that

$$\begin{aligned}2\|FD\|_{L^2} \leq&\|P\|_{L^2}^2\|H\|_{L^2}\|\Gamma_3\|_{L^2}\|H\|_{L^1}T^{\frac{1}{2}}+|a|T^{\frac{1}{2}}\\ &\|P\|_{L^2}\|H\|_{L^2}\|\Gamma_3\|_{L^2}\|V\|_{L^1}\|D\|_{L^2}\\ &+\|P\|_{L^2}\|H\|_{L^2}\|P\|_{L^2}\|S\|_{L^2}T^{\frac{1}{2}}\\ &+|a|\|P\|_{L^2}\|H\|_{L^2}\|K\|_{L^2}\|D\|_{L^2}T^{\frac{1}{2}}\\ &+|a|\|D\|_{L^2}\|V\|_{L^2}\|\Gamma_3\|_{L^2}\|H\|_{L^1}T^{\frac{1}{2}}\\ &+a^2\|D\|_{L^2}^2\|V\|_{L^2}\|\Gamma_3\|_{L^2}\|V\|_{L^1}T^{\frac{1}{2}}\\ &+|a|\|D\|_{L^2}\|V\|_{L^2}T^{\frac{1}{2}}\|P\|_{L^2}\|S\|_{L^2}\\ &+a^2\|D\|_{L^2}^2\|V\|_{L^2}\|K\|_{L^2}T^{\frac{1}{2}},\end{aligned} \tag{2}$$

which implies that $\|FD\|_{L^2} \leq \infty$. On the other hand, we notice that $[((P*H)(t)+a(D*V)(t)]^{+} \geq 0$ and $([P*H+aD*V]^{-}*\Gamma_{n_3,\tau_3})(t) \geq 0$, it easily follows that $F(L^2([0,T],R_+)) \subset L^2([0,T],R_+)$. The proof is completed.

The Proof of Theorem 4. By equation (1) and the properties of absolute value $|\cdot|$, we have

$$\begin{aligned}2|(FD)(t)|\leq&|(P*H)(t)||P*H|*\Gamma_3(t)|+|(P*H)(t)|\\ &\times||aD*V|*\Gamma_3(t)|+|(P*H)(t)|\\ &\times|(P*S)(t)|+|(P*H)(t)||a(D*K)(t)|\\ &+|a(D*V)(t)||(P*S)(t)|+|a(D*V)(t)|\\ &\times|a(D*K)(t)|+|a(D*V)(t)|||(P*H)|\\ &*\Gamma_3(t)|+|a(D*V)(t)|||aD*V|*\Gamma_3(t)|.\end{aligned}$$

The Proof of Theorem 5. Let $\{D_n\}$ and $D_0 \in L^2([0,T],R_+)$ such that $\|D_n - D_0\|_{L^2} \to 0$ as $n \to \infty$. By applying equation (1) and the properties of absolute value $|\cdot|$, we obtain

$$\begin{aligned}&|(FD_n)(t)-(FD_0)(t)|\\ \leq&|([P*H+aD_n*V]^{-}-[P*H+aD_0*V]^{-})\\ &*\Gamma_3(t)\times[P*H(t)+aD_0*V(t)]^{+}|\\ &+|([P*H(t)+aD_n*V(t)]^{+}-[P*H(t)\\ &+aD_0*V(t)]^{+})\times([P*H+aD_n*V]^{-}*\Gamma_3(t))|.\end{aligned}$$

By using $f^+ = (|f|+f)/2$ and $f^- = (|f|-f)/2$, we have

$$\begin{aligned}&2|(FD_n)(t) - (FD_0)(t)|\\ \le&(|(|a(D_n - D_0) * V|*\Gamma_3)(t)|+|a(D_n - D_0) * K(t)|)\\ &\times (|P * H(t)|+|aD_0 * V(t)|) + (|a(D_n - D_0) * V(t)|)\\ &\times (||P * H|*\Gamma_3(t)|+||aD_n * V|*\Gamma_3(t)|). \end{aligned} \quad (3)$$

Using Hölder's inequality and (H_1), we get

$$\begin{aligned}&\|FD_n - FD_0\|_{L^2}\\ \le&\big(|a|\|V\|_{L^2}\|\Gamma_3\|_{L^2}\|P\|_{L^2}\|H\|_{L^1}T^{\frac{1}{2}} + a^2\|V\|_{L^2}\\ &\times \|\Gamma_3\|_{L^2}\|D_n\|_{L^2}\|V\|_{L^1}T^{\frac{1}{2}} + \frac{1}{2}|a|\|P\|_{L^2}\|H\|_{L^2}\\ &\times \|\Gamma_3\|_{L^2}\|V\|_{L^1}T^{\frac{1}{2}} + \frac{1}{2}|a|\|P\|_{L^2}\|H\|_{L^2}\|K\|_{L^2}T^{\frac{1}{2}}\\ &+ \frac{1}{2}a^2\|D_0\|_{L^2}\|V\|_{L^2}\|\Gamma_3\|_{L^2}\|V\|_{L^1}T^{\frac{1}{2}}\\ &+ \frac{1}{2}a^2\|D_0\|_{L^2}\|V\|_{L^2}\|K\|_{L^2}T^{\frac{1}{2}}\big) \times \|D_n - D_0\|_{L^2}\\ =&Q\|D_n - D_0\|_{L^2}, \end{aligned} \quad (4)$$

which by (4) yields that

$$\lim_{n\to\infty} \|FD_n - FD_0\|_{L^2} = 0. \quad (5)$$

Therefore F is continuous. The proof is completed.

THE PROOF OF THEOREM 6. To show the compactness of operator F, it only needs to prove that the operator F maps bounded sets S of $L^2([0,T], R_+)$ into a sequentially compact set. It means that we need to prove $F(S)$ is a sequentially compact set. Let S be a bounded set of $L^2([0,T], R_+)$, there exists a real number $M > 0$ such that $\|D\|_{L^2} \le M$, for any $D \in S$. We will derive $F(S)$ is a sequentially compact set in the following two steps. Firstly, we prove $F(S)$ is bounded. It follows from the (1) that

$$\begin{aligned}2\|FD\|_{L^2} \le&\|P\|^2_{L^2}\|H\|_{L^2}\|\Gamma_3\|_{L^2}\|H\|_{L^1}T^{\frac{1}{2}} + |a|T^{\frac{1}{2}}\|P\|_{L^2}\\ &\times \|H\|_{L^2}\|\Gamma_3\|_{L^2}\|V\|_{L^1}\|D\|_{L^2} + \|P\|_{L^2}\|H\|_{L^2}\\ &\times \|P\|_{L^2}\|S\|_{L^2}T^{\frac{1}{2}} + |a|\|P\|_{L^2}\|H\|_{L^2}\|K\|_{L^2}\\ &\times \|D\|_{L^2}T^{\frac{1}{2}} + |a|\|D\|_{L^2}\|V\|_{L^2}\|\Gamma_3\|_{L^2}T^{\frac{1}{2}}\\ &\times \|H\|_{L^1} + a^2\|D\|^2_{L^2}\|V\|_{L^2}\|\Gamma_3\|_{L^2}\|V\|_{L^1}T^{\frac{1}{2}}\\ &+ |a|\|D\|_{L^2}\|V\|_{L^2}\|P\|_{L^2}\|S\|_{L^2}T^{\frac{1}{2}}\\ &+ a^2\|D\|^2_{L^2}\|V\|_{L^2}\|K\|_{L^2}T^{\frac{1}{2}}\\ <&\infty, \end{aligned} \quad (6)$$

thus establishing condition (i) of Theorem 1. Secondly, we prove condition (ii) of Theorem 1. Now, let $\delta > 0$ and $0 < h < \delta$, we have

$$\begin{aligned}&|(FD)(t+h) - (FD)(t)|\\ \le&\frac{1}{2}\big(|P * (H(t+h) - H(t))|+|aD * (V(t+h) - V(t))|\big)\\ &\times \big(|aD * K(t+h)|+|(|P * H|*\Gamma_3(t+h)|\\ &+ |P * S(t+h)|+||aD * V|*\Gamma_3(t+h)|\big)\\ &+ \frac{1}{2}\big(2|(P * H)|*(\Gamma_3(t+h) - \Gamma_3(t))|\\ &+ ||aD * V|*(\Gamma_3(t+h) - \Gamma_3(t))|\\ &+ |aD * (K(t+h) - K(t))|\big)\\ &\times \big(|(P * H)(t)|+|a(D * V)(t)|\big)\\ =&I_1 + I_2, \end{aligned} \quad (7)$$

for a.e. $t \in [0,T]$, where

$$\begin{aligned}2I_1 =& \big(|(|P * H|*\Gamma_3)(t+h)|+|(P * S)(t+h)|\\ &+ |(|aD * V|*\Gamma_3)(t+h)|+|a(D * K)(t+h)|\big)\\ &\times |P * (H(t+h) - H(t))|+\big(|(P * S)(t+h)|\\ &+ |(|P * H|*\Gamma_3)(t+h)|+|a(D * K)(t+h)|\\ &+ |(|aD * V|*\Gamma_3)(t+h)|\big)\\ &\times |aD * (V(t+h) - V(t))|. \end{aligned} \quad (8)$$

and

$$\begin{aligned}I_2 =&|P * H||*(\Gamma_3(t+h) - \Gamma_3)(t)|\big(|(P * H)(t)|\\ &+ |a(D * V)(t)|\big) + \frac{1}{2}\big(|(P * H)(t)|+|a(D * V)(t)|\big)|\\ &\times ||aD * V|*(\Gamma_3(t+h) - \Gamma_3(t))|\\ &+ \frac{1}{2}\big(|(P * H)(t)|+|a(D * V)(t)|\big)\\ &\times |aD * (K(t+h) - K(t))|. \end{aligned}$$

Let us first discuss the I_1. By applying Hölder's inequality and (H_1), we deduce that

$$\begin{aligned}\|I_1\|_{L^2} \le&\frac{1}{2}\big(\|\Gamma_3\|_{L^2}\|P\|_{L^2}\|H\|_{L^1}\|P\|_{L^2}T^{\frac{1}{2}} + |a|T^{\frac{1}{2}}\|V\|_{L^1}\\ &\times \|\Gamma_3\|_{L^2}\|D\|_{L^2}\|P\|_{L^2} + \|P\|^2_{L^2}T^{\frac{1}{2}}\|S\|_{L^2}\\ &+ |a|\|D\|_{L^2}\|K\|_{L^2}\|P\|_{L^2}T^{\frac{1}{2}}\big)\\ &\times \Big(\int_R |H(t+h) - H(t)|^2 dt\Big)^{\frac{1}{2}}\\ &+ \frac{1}{2}\big(|a|\|\Gamma_3\|_{L^2}\|P\|_{L^2}\|H\|_{L^1}\|D\|_{L^2}T^{\frac{1}{2}} + a^2T^{\frac{1}{2}}\\ &\times \|\Gamma_3\|_{L^2}\|D\|^2_{L^2}\|V\|_{L^1} + |a|\|P\|_{L^2}\|S\|_{L^2}T^{\frac{1}{2}}\\ &\times \|D\|_{L^2} + a^2\|D\|^2_{L^2}\|K\|_{L^2}T^{\frac{1}{2}}\big)\\ &\times \Big(\int_R |V(t+h) - V(t)|^2 dt\Big)^{\frac{1}{2}}. \end{aligned} \quad (9)$$

Equation (9) can be expressed as

$$\|I_1\|_{L^2} \leq A_1\Big(\int_R |H(t+h)-H(t)|^2dt\Big)^{\frac{1}{2}} + B_1\Big(\int_R |V(t+h)-V(t)|^2dt\Big)^{\frac{1}{2}}. \tag{10}$$

Now, we discuss I_2 in a same way. By applying Hölder's inequality, we get that

$$\begin{aligned}\|I_2\|_{L^2} \leq &(\|P\|_{L^2}\|H\|_{L^2}\|P\|_{L^2}\|H\|_{L^1}T^{\frac{1}{2}} + |a|\|D\|_{L^2}T^{\frac{1}{2}}\\ &\times \|V\|_{L^2}\|H\|_{L^1}\|P\|_{L^2} + \frac{1}{2}|a|\|P\|_{L^2}\|H\|_{L^2}T^{\frac{1}{2}}\\ &\times \|D\|_{L^2}\|V\|_{L^1} + \frac{1}{2}a^2\|D\|^2_{L^2}\|V\|_{L^2}\|V\|_{L^1}T^{\frac{1}{2}})\\ &\times \Big(\int_R |\Gamma_3(t+h)-\Gamma_3(t)|^2dt\Big)^{\frac{1}{2}} + \frac{1}{2}\\ &\times \big(\|P\|_{L^2}\|H\|_{L^2}\|D\|_{L^2}T^{\frac{1}{2}} + \|D\|^2_{L^2}\|V\|_{L^2}T^{\frac{1}{2}}\big)\\ &\times \Big(\int_R |K(t+h)-K(t)|^2dt\Big)^{\frac{1}{2}}.\end{aligned} \tag{11}$$

Equation (11) can be expressed as

$$\|I_2\|_{L^2} \leq A_2\Big(\int_R |\Gamma_3(t+h)-\Gamma_3(t)|^2dt\Big)^{\frac{1}{2}} + B_2\Big(\int_R |K(t+h)-K(t)|^2dt\Big)^{\frac{1}{2}}. \tag{12}$$

It follows from the (12) and (10) that

$$\begin{aligned}&\|(FD)(\cdot+h)-(FD)(\cdot)\|_{L^2}\\ \leq &A_1\Big(\int_R |H(t+h)-H(t)|^2dt\Big)^{\frac{1}{2}} + B_1\Big(\int_R |V(t+h)\\ &-V(t)|^2dt\Big)^{\frac{1}{2}} + A_2\Big(\int_R |\Gamma_3(t+h)-\Gamma_3(t)|^2dt\Big)^{\frac{1}{2}}\\ &+ B_2\Big(\int_R |K(t+h)-K(t)|^2dt\Big)^{\frac{1}{2}},\end{aligned} \tag{13}$$

where A_1, A_2, B_1, B_2 are positive real numbers. In order to prove that condition (ii) of Theorem 1, we discuss the right of the equation (13). Since $C_c(R)$ is dense in $L^2(R)$. Therefore for any $H(t) \in L^2(R)$ and $\epsilon > 0$ there exists a function $g \in C_c(R)$ such that $\|H-g\|_{L^2} \leq \frac{\epsilon}{12A_1}$. Applying continuity of function $g(t)$, it follows that we can choose $\delta_1 > 0$ with $0 < h < \delta_1$ such that $(\int_R |g(t+h) - g(t)|^2dt)^{\frac{1}{2}} < \frac{\epsilon}{12A_1}$. Therefore for $0 < h < \delta_1$, we have

$$\begin{aligned}&\|H(\cdot+h)-H(\cdot)\|_{L^2}\\ \leq &\Big(\int_R |H(t+h)-g(t+h)|^2dt\Big)^{\frac{1}{2}} + \Big(\int_R |g(t+h)\\ &-g(t)|^2dt\Big)^{\frac{1}{2}} + \Big(\int_R |g(t)-H(t)|^2dt\Big)^{\frac{1}{2}}\\ < &\frac{\epsilon}{12A_1}\times 3 = \frac{\epsilon}{4A_1}.\end{aligned} \tag{14}$$

In same way, we can choose δ_2, δ_3 and $\delta_4 > 0$ with $0 < h < \delta_2$, $0 < h < \delta_3$ and $0 < h < \delta_4$ such that $(\int_R |V(t+h) - V(t)|^2dt)^{\frac{1}{2}} < \frac{\epsilon}{4B_1}$, $(\int_R |\Gamma_3(t+h) - \Gamma_3(t)|^2dt)^{\frac{1}{2}} < \frac{\epsilon}{4A_2}$ and $(\int_R |K(t+h) - K(t)|^2dt)^{\frac{1}{2}} < \frac{\epsilon}{4B_2}$. Therefore we can take $\delta = \min\{\delta_1, \delta_2, \delta_3, \delta_4\}$ with $0 < h < \delta$ such that

$$\Big(\int_{[0,T]} |(FD)(t+h)-(FD)(t)|^2dt\Big)^{\frac{1}{2}} < \epsilon. \tag{15}$$

Thanks to (15), it is clear that condition (ii) of Theorem 1 is satisfied. Further, it follows from the Theorem 1 that $F(S)$ is a sequentially compact set. The proof is completed.

THE PROOF OF THEOREM 7. Let r be a positive real number and consider the closed, convex and bounded set B_r of L^2, which is defined by

$$B_r = \{D \in L^2([0,T], R_+), \|D\|_{L^2} \leq r\}.$$

For any $D \in B_r$. From (1), it immediately follows that

$$\begin{aligned}|(FD)(t)| \leq &\frac{1}{2}\big(|P * S(t)| + ||P*H|*\Gamma_3(t)| + |aD*K(t)|\\ &+ ||aD*V|*\Gamma_3(t)|\big) \times \big(|(P*H)(t)|\\ &+ |a(D*V)(t)|\big).\end{aligned} \tag{16}$$

By simple calculation, we deduce that

$$\begin{aligned}\|FD\|_{L^2} \leq &\frac{1}{2}\|P\|^2_{L^2}\|H\|_\infty\|S\|_\infty T^{\frac{3}{2}} + \frac{1}{2}\|P\|^2_{L^2}\|H\|_\infty T^{\frac{3}{2}}\\ &\times \|H\|_{L^1}\|\Gamma_3\|_\infty - \frac{a}{2}(\|P\|_{L^2}\|H\|_\infty\|K\|_\infty T^{\frac{3}{2}}\\ &+ \|P\|_{L^2}\|H\|_\infty\|\Gamma_3\|_{L^2}\|V\|_{L^1}T + \|P\|_{L^2}T^{\frac{3}{2}}\\ &\times \|S\|_\infty\|V\|_\infty + \|P\|_{L^2}\|H\|_{L^1}\|\Gamma_3\|_\infty\|V\|_\infty\\ &\times T^{\frac{3}{2}})\|D\|_{L^2} + \frac{a^2}{2}(\|V\|_\infty T\|V\|_{L^1}\|\Gamma_3\|_{L^2}\\ &+ \|V\|_\infty\|K\|_\infty T^{\frac{3}{2}})\|D\|^2_{L^2}.\end{aligned}$$

Let $N' = \frac{1}{2}\|V\|_\infty\|V\|_{L^1}T\|\Gamma_3\|_{L^2} + \frac{1}{2}\|V\|_\infty T^{\frac{3}{2}}\|K\|_\infty$, $\varphi = \frac{1}{2}\|P\|^2_{L^2}\|H\|_\infty\|S\|_\infty T^{\frac{3}{2}} + \frac{1}{2}\|P\|^2_{L^2}\|H\|_\infty\|H\|_{L^1}\|\Gamma_3\|_\infty T^{\frac{3}{2}}$, and $Q = \frac{1}{2}(\|P\|_{L^2}\|H\|_\infty\|K\|_\infty T^{\frac{3}{2}} + \|P\|_{L^2}\|H\|_\infty\|V\|_{L^1} \|\Gamma_3\|_{L^2}T + \|P\|_{L^2}\|S\|_\infty\|V\|_\infty T^{\frac{3}{2}} + \|P\|_{L^2}\|H\|_{L^1} T^{\frac{3}{2}}\|\Gamma_3\|_\infty\|V\|_\infty)$, then we obtain

$$\|(FD)\|_{L^2} \leq \varphi - aQ\|D\|_{L^2} + a^2N'\|D\|^2_{L^2}. \tag{17}$$

Now, we consider the equation

$$\varphi - aQr + a^2N'r^2 \leq r. \tag{18}$$

has solutions. Let

$$b^2 - 4ac = a^2(Q^2 - 4N'\varphi) + 2aQ + 1 \geq 0. \tag{19}$$

In order to get our conclusions, we need to discuss three cases. We first consider the following case1:

Case1: If $Q^2 - 4N'\varphi = 0$, we derive from (19) that $a \in \big[-\frac{1}{2Q}, 0\big)$. Furthermore, by plugging a into (18), we get

$$\frac{(aQ+1) - \sqrt{2aQ+1}}{2a^2N'} \le r \le \frac{(aQ+1) + \sqrt{2aQ+1}}{2a^2N'}. \tag{20}$$

Moreover, when $a \in \big[-\frac{1}{2Q}, 0\big)$, we have

$$\frac{(aQ+1) - \sqrt{2aQ+1}}{2a^2N'} \ge 0. \tag{21}$$

Therefore, we conclude that $F(B_r) \subset B_r$. By applying Theorem 3, we can get that the equation (1) has at least one solution on $B_r = \big\{D \in L^2([0,T], R_+), \|D\|_{L^2} \le r\big\}$. It is clear that condition (i) of Theorem 7 is satisfied.

Now, we consider the case2:

Case2: If $Q^2 - 4N'\varphi > 0$, by using (19), we easily get $a \in \big[\frac{-1}{Q - 2\sqrt{N\varphi}}, \frac{-1}{Q + 2\sqrt{N\varphi}}\big]$. Furthermore, applying $aQ + 1 > 0$, we get $a \in \big(-\frac{1}{Q}, 0\big)$. Using $a \in \big[\frac{-1}{Q - 2\sqrt{N\varphi}}, \frac{-1}{Q + 2\sqrt{N\varphi}}\big]$ and together with $a \in \big[-\frac{1}{Q}, 0\big)$, we can get $a \in \big(-\frac{1}{Q}, \frac{-1}{Q + 2\sqrt{N\varphi}}\big]$. By substituting a into (18), we have

$$\begin{aligned}\frac{(aQ+1) - \sqrt{(aQ+1)^2 - 4a^2N'\varphi}}{2a^2N'} &\le \\ r \le \frac{(aQ+1) + \sqrt{(aQ+1)^2 - 4a^2N'\varphi}}{2a^2N'}&.\end{aligned} \tag{22}$$

It follows from (18) that $F(B_r) \subset B_r$. Based on Theorem 3, we can get that the equation (1) has solutions on $B_r = \big\{D \in L^2([0,T], R_+), \|D\|_{L^2} \le r\big\}$. Therefore, the condition (ii) of Theorem 7 is satisfied.

Finally, we consider the case3:

Case3: If $Q^2 - 4N'\varphi < 0$ and $aQ + 1 > 0$, we derive from (19) that $a \in \big[\frac{-1}{Q + 2\sqrt{N\varphi}}, 0\big)$. Furthermore, by applying (18), we get

$$\begin{aligned}\frac{(aQ+1) - \sqrt{(aQ+1)^2 - 4a^2N'\varphi}}{2a^2N'} &\le \\ r \le \frac{(aQ+1) + \sqrt{(aQ+1)^2 - 4a^2N'\varphi}}{2a^2N'}&.\end{aligned} \tag{23}$$

It follows from (18) that $F(B_r) \subset B_r$. Based on Theorem 3, we can get that the equation (1) has at least one solution on $B_r = \big\{D \in L^2([0,T], R_+), \|D\|_{L^2} \le r\big\}$. Therefore, the condition (iii) of Theorem 7 is satisfied. The proof is completed.

THE PROOF OF THEOREM 8. (i). Suppose that exists two elements $D_1(t), D_2(t) \in B_r$ such that $D_1 = FD_1$ and $D_2 = FD_2$. For a.e. $t \in [0,T]$, using the definition of positive and negative, we have

$$\begin{aligned}FD_1 - FD_2 =&([P*H + aD_1*V]^- * \Gamma_3)(t) \times [P*H(t) \\ &+ aD_1*V(t)]^+ - ([P*H + aD_2*V]^- \\ &* \Gamma_3)(t) \times [P*H(t) + aD_2*V(t)]^+ \\ =&\frac{1}{4}(|P*H + aD_1*V| - (P*H + aD_1*V)) \\ &* \Gamma_3(t) \times (|P*H + aD_1*V| + P*H \\ &+ aD_1*V) - \frac{1}{4}(|P*H + aD_2*V| \\ &- (P*H + aD_2*V)) * \Gamma_3(t) \\ &\times (|P*H + aD_2*V| + P*H + aD_2*V).\end{aligned}$$

Moreover, applying the properties of absolute value $|\cdot|$, we have

$$\begin{aligned}&|FD_1 - FD_2| \\ \le&\frac{1}{2}(||a(D_1 - D_2)*V|*\Gamma_3(t)| + |a(D_1 - D_2)*K|) \\ &\times (|P*H| + |aD_1*V|) + \frac{1}{2}|a(D_1 - D_2)*V| \\ &\times (||P*H|*\Gamma_3(t)| + ||aD_2*V|*\Gamma_3(t)| \\ &+ |P*S| + |aD_2*K|).\end{aligned}$$

Applying the Hölder's inequality and (H_1), the following inequality holds:

$$\begin{aligned}&\|FD_1 - FD_2\|_{L^2} \\ \le&\Big(a^2r(\|V\|_\infty\Gamma_3\|_{L^2}\|V\|_{L^1}T + \|K\|_\infty\|V\|_\infty T^{\frac{3}{2}}) \\ &- a(\frac{1}{2}\|P\|_{L^2}\|H\|_\infty\Gamma_3\|_{L^2}\|V\|_{L^1}T + \frac{1}{2}\|P\|_{L^2} \\ &\|H\|_\infty\|K\|_\infty T^{\frac{3}{2}} + \frac{1}{2}\|P\|_{L^2}\|H\|_{L^1}\|\Gamma_3\|_\infty\|V\|_\infty \\ &T^{\frac{3}{2}} + \frac{1}{2}\|P\|_{L^2}\|S\|_\infty\|V\|_\infty T^{\frac{3}{2}})\Big)\|D_1 - D_2\|_{L^2}.\end{aligned} \tag{24}$$

In view of $Q^2 - 4N'\varphi = 0$ and $a \in [-\frac{1}{2Q}, 0)$, we derive from (24) that

$$\|FD_1 - FD_2\|_{L^2} =(-aQ + 2a^2N'r)\|D_1 - D_2\|_{L^2}, \tag{25}$$

where $0 \leq (-aQ + 2a^2N'r) < 1$. This implies that F is contraction. (ii). If $Q^2 - 4N'\varphi < 0$ and for any $a \in [\frac{-1}{Q+2\sqrt[2]{N'\varphi}}, 0)$, from (24), we have

$$\|FD_1 - FD_2\|_{L^2} \leq (-aQ + 2a^2N'r)\|D_1 - D_2\|_{L^2} \tag{26}$$

and $0 \leq (-aQ + 2a^2N'r) < 1$, which leads to the operator F is contraction. Consequently, we get $D_1 = D_2$. The proof is completed.
