# Supplementary material for: Mathematical study of neural feedback roles in small target motion detection
Source: Front Neurorobot. 2022 Sep 20;16:984430. doi: 10.3389/fnbot.2022.984430 (PMC9530796; doi:10.3389/fnbot.2022.984430)
Supplement: Supplementary file 2 [file Data_Sheet_1.ZIP › Supplementary Material Presentation/Fig11-eps-converted-to.pdf]

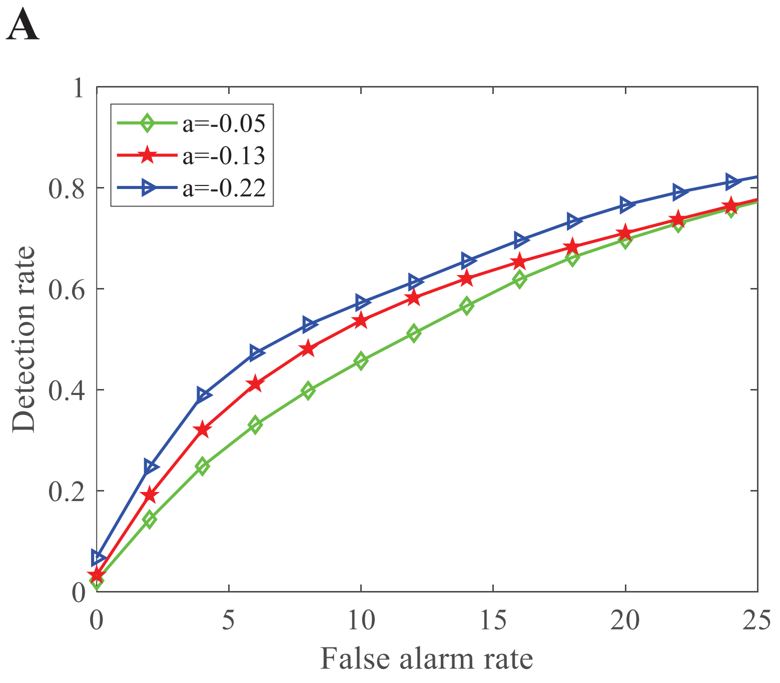

The detection rate for different feedback constant a

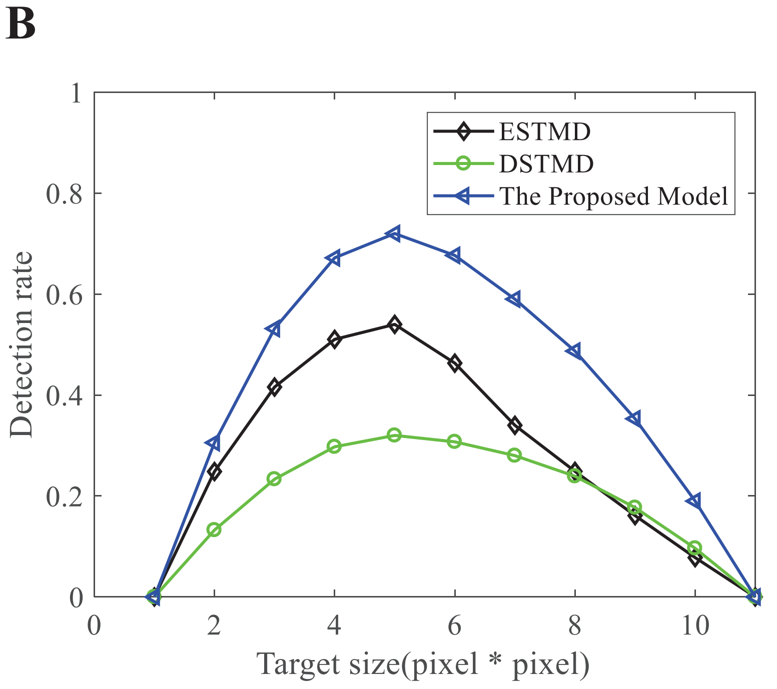

The detection rate for different target size

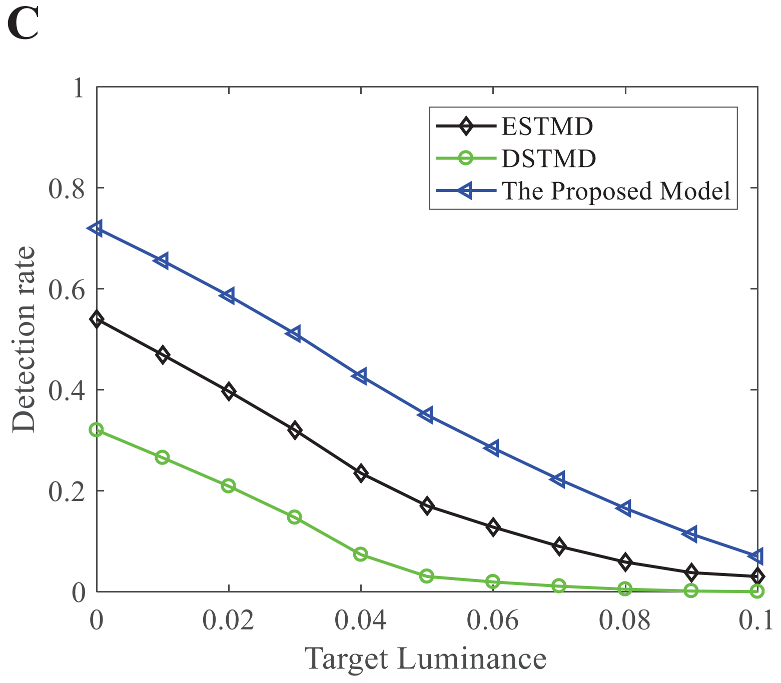

The detection rate for different target luinance

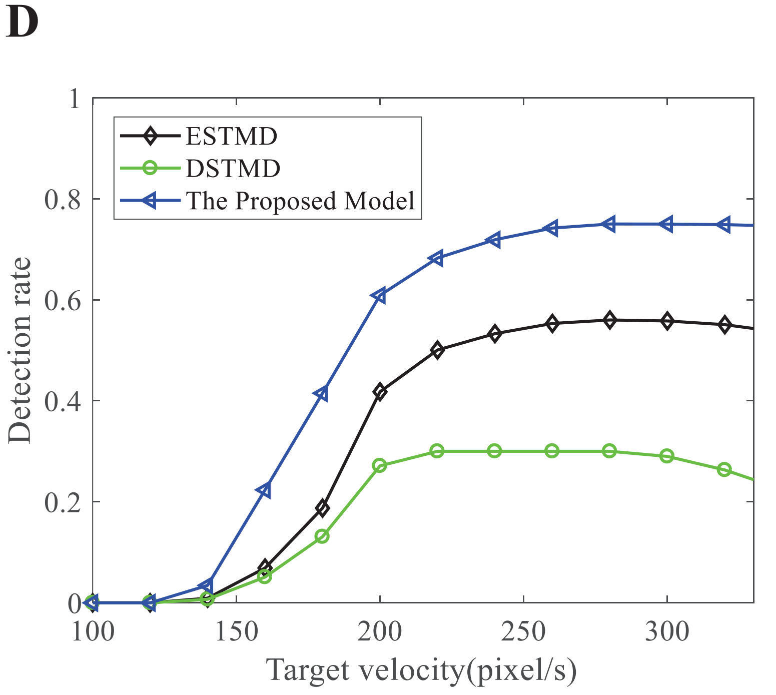

The detection rate for different target velocity

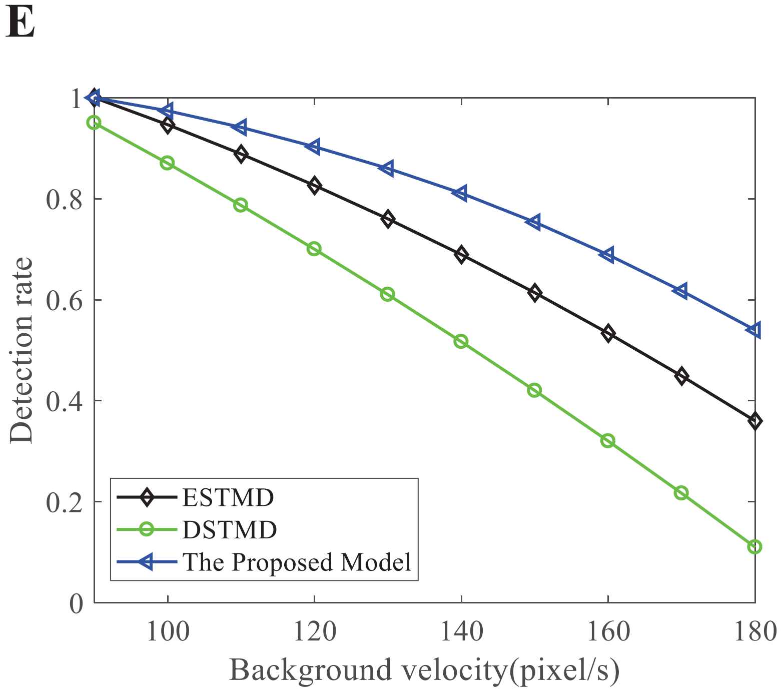

The detection rate for different background velocity

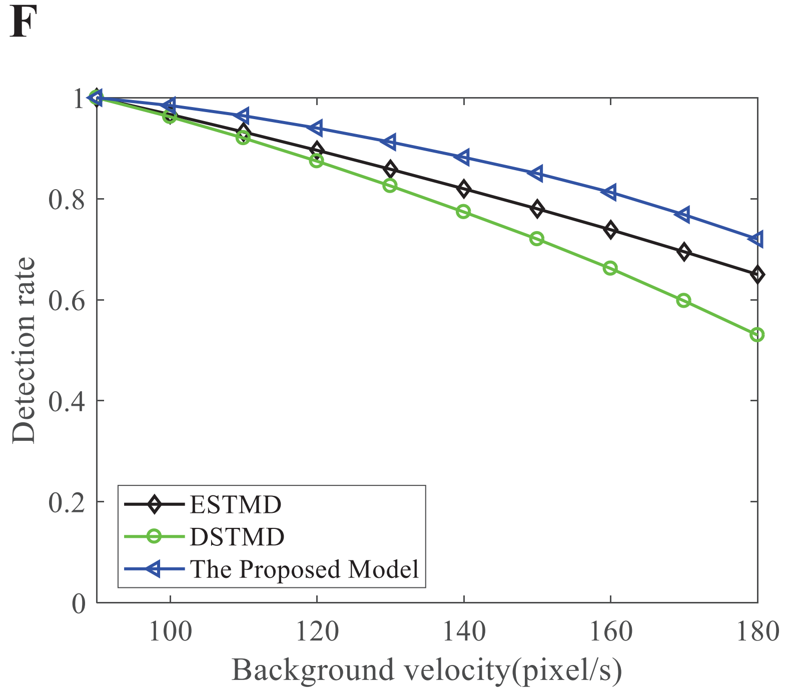

The detection rate for different background velocity
