# Supplementary material for: Mathematical study of neural feedback roles in small target motion detection
Source: Front Neurorobot. 2022 Sep 20;16:984430. doi: 10.3389/fnbot.2022.984430 (PMC9530796; doi:10.3389/fnbot.2022.984430)
Supplement: Supplementary file 2 [file Data_Sheet_1.ZIP › Supplementary Material Presentation/Fig7-eps-converted-to.pdf]

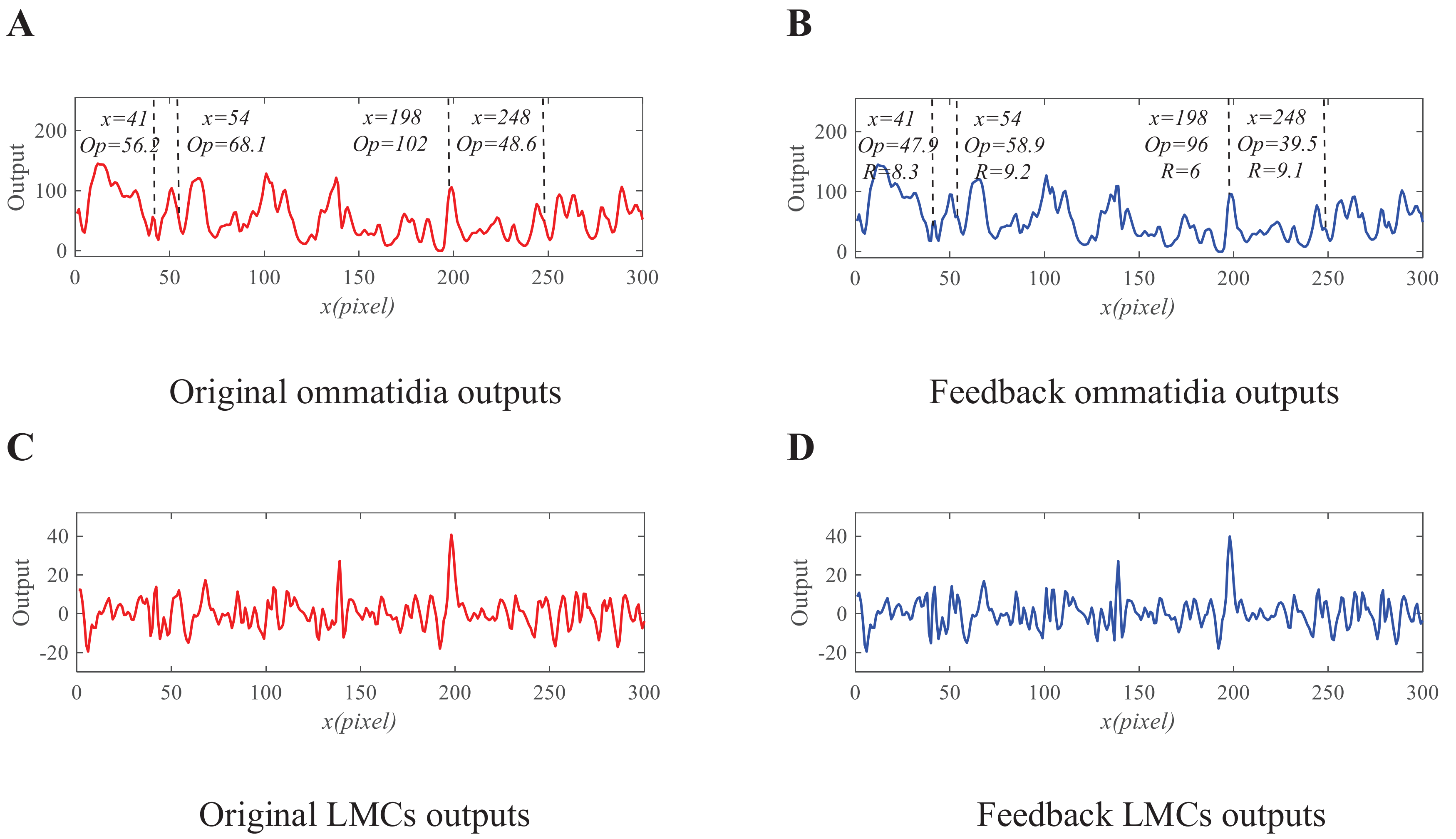
A
x=41
Op=56.2
x=54
Op=68.1
x=198
Op=102
x=248
Op=48.6
Output
x(pixel)
Original ommatidia outputs
B
x=41
Op=47.9
R=8.3
x=54
Op=58.9
R=9.2
x=198
Op=96
R=6
x=248
Op=39.5
R=9.1
Output
x(pixel)
Feedback ommatidia outputs
C
Output
x(pixel)
Original LMCs outputs
D
Output
x(pixel)
Feedback LMCs outputs
