# Supplementary material for: Mathematical study of neural feedback roles in small target motion detection
Source: Front Neurorobot. 2022 Sep 20;16:984430. doi: 10.3389/fnbot.2022.984430 (PMC9530796; doi:10.3389/fnbot.2022.984430)
Supplement: Supplementary file 2 [file Data_Sheet_1.ZIP › Supplementary Material Presentation/Fig9-eps-converted-to.pdf]

A

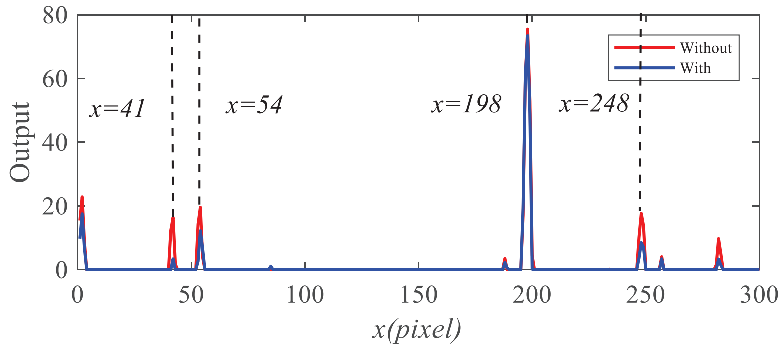

The original STMDs outputs and feedback outputs

B

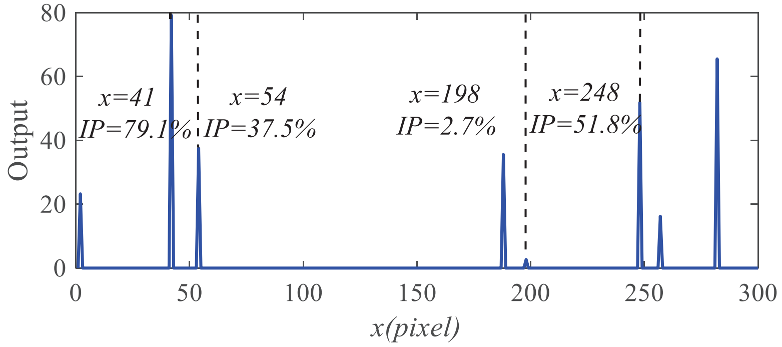

The inhibition percentage (IP) of the different locations
