# Supplementary figures and images for: Mathematical study of neural feedback roles in small target motion detection
Source: Front Neurorobot. 2022 Sep 20;16:984430. doi: 10.3389/fnbot.2022.984430 (PMC9530796; doi:10.3389/fnbot.2022.984430)

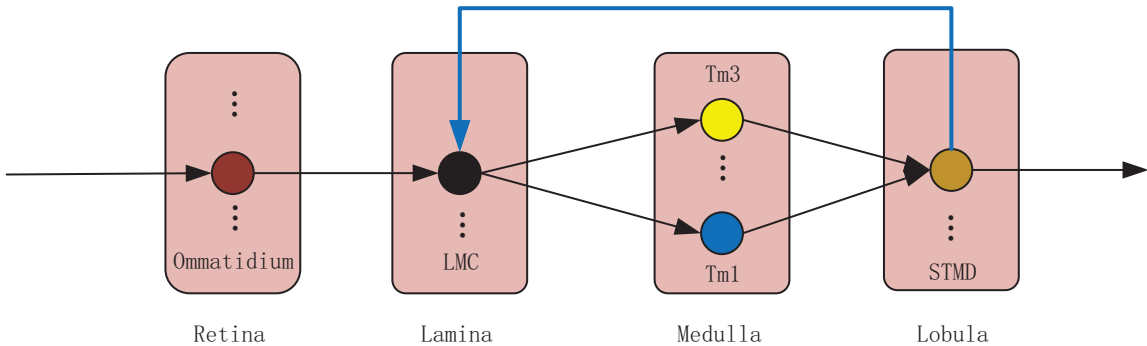

Supplement: Supplementary file 2 [file Data_Sheet_1.ZIP › Supplementary Material Presentation/Fig1-eps-converted-to.pdf]

**A**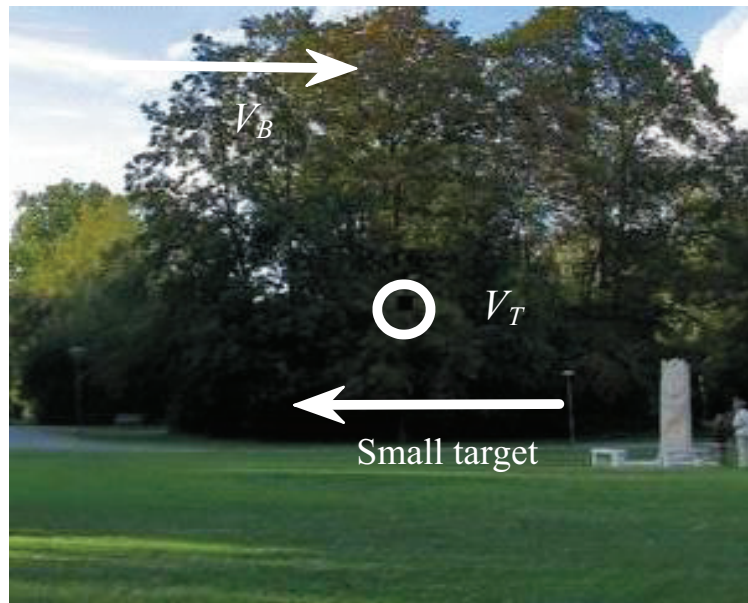

A frame of the initial sequence

**B**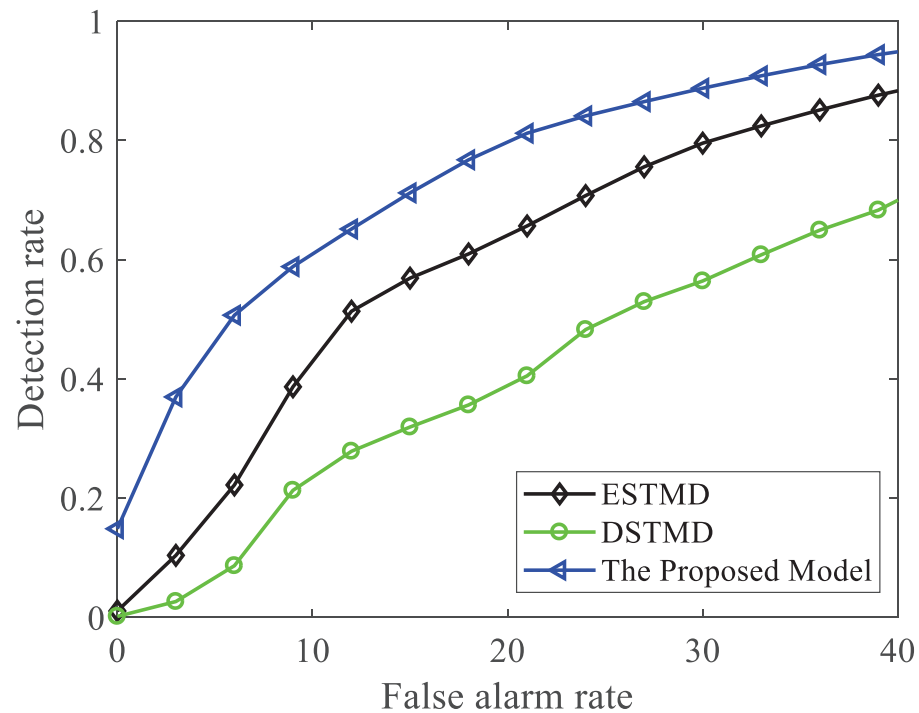

The detection rate curves of the initial image sequence

Supplement: Supplementary file 2 [file Data_Sheet_1.ZIP › Supplementary Material Presentation/Fig10-eps-converted-to.pdf]

**A**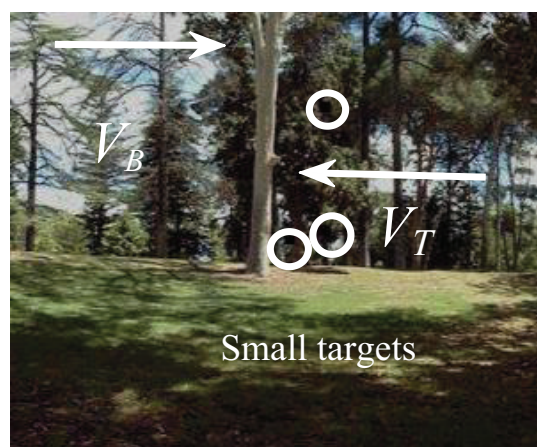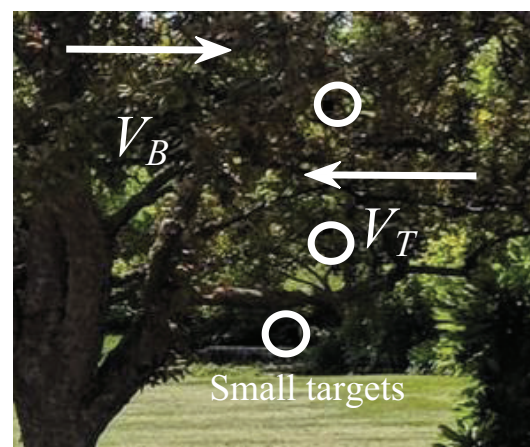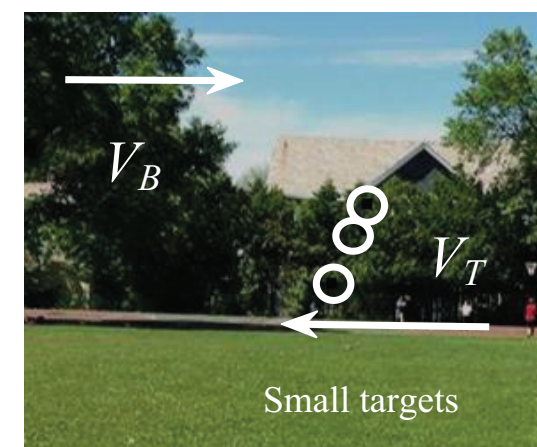**B**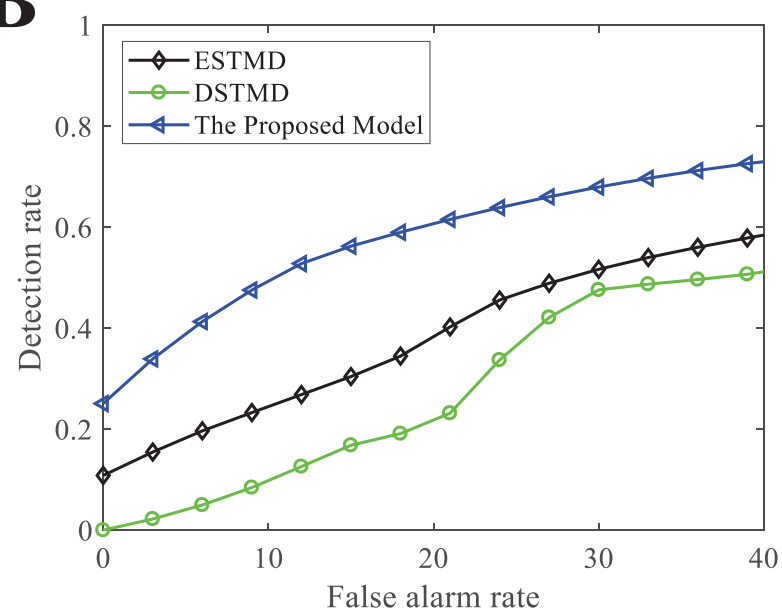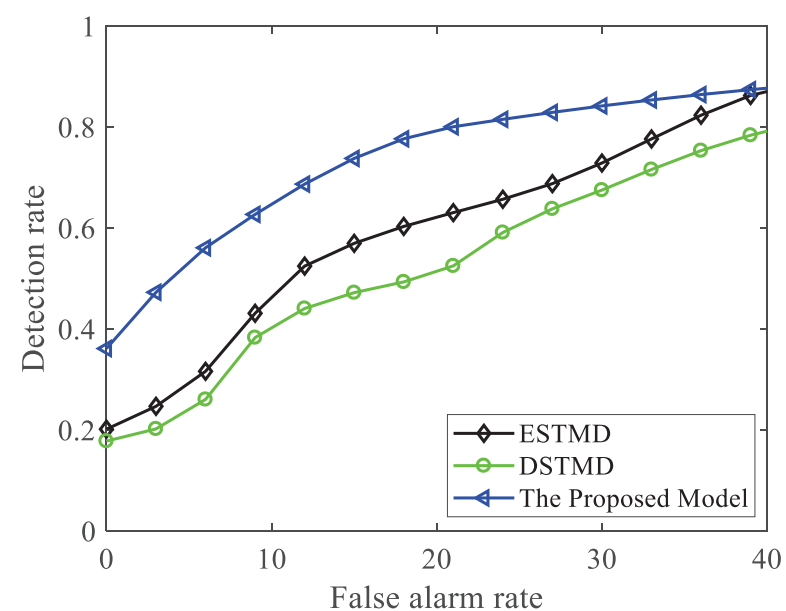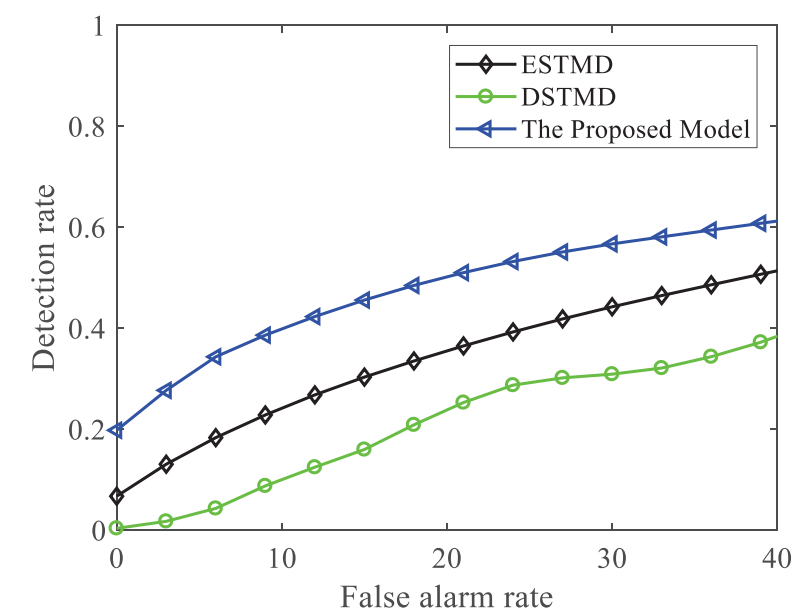**C**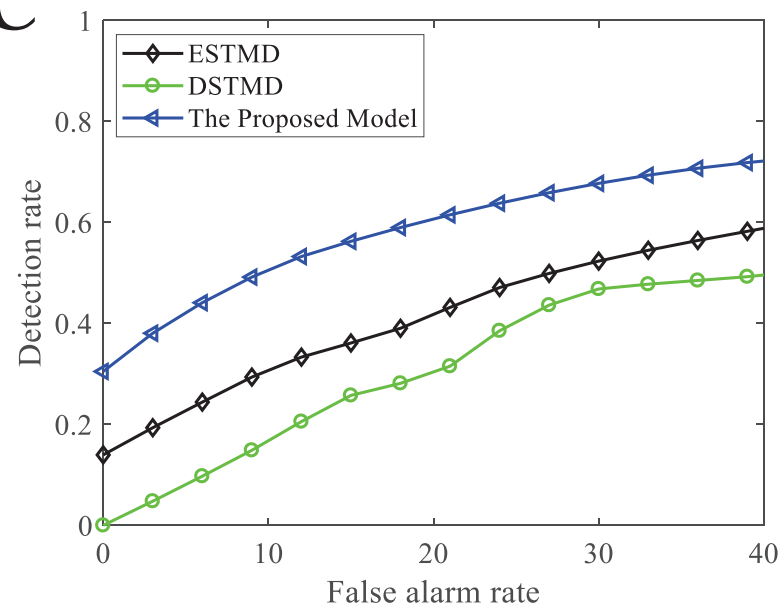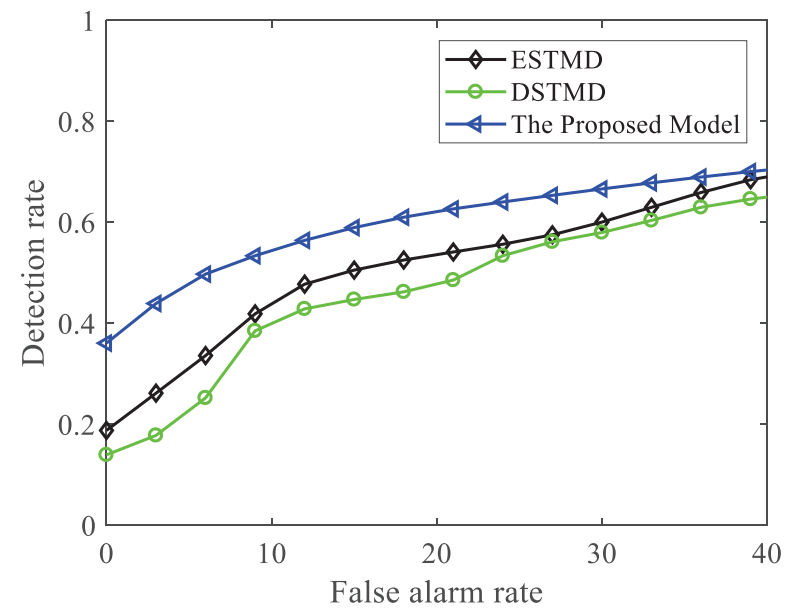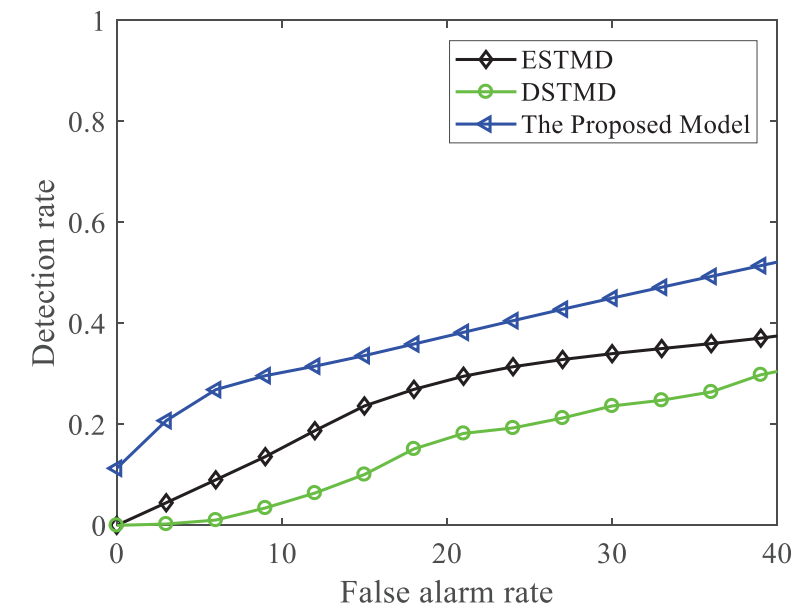

Supplement: Supplementary file 2 [file Data_Sheet_1.ZIP › Supplementary Material Presentation/Fig12-eps-converted-to.pdf]

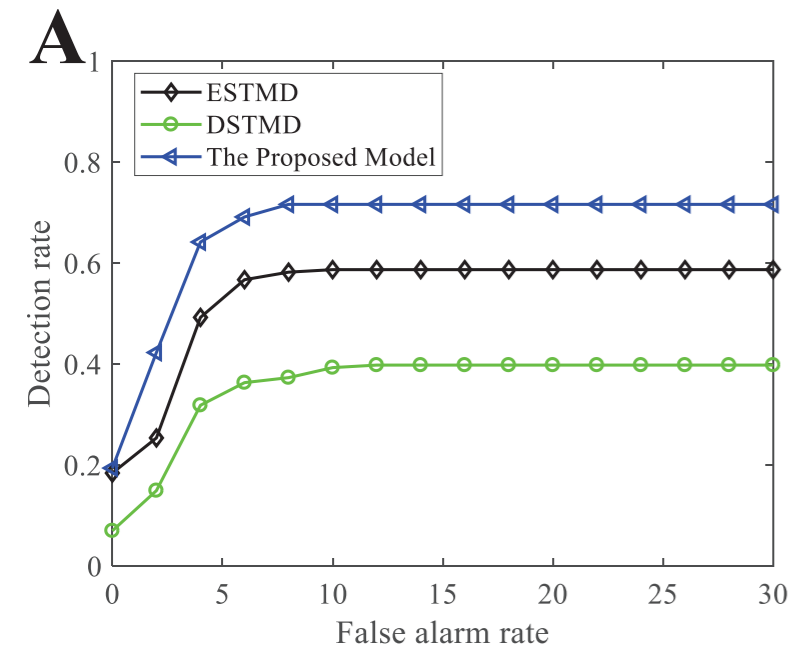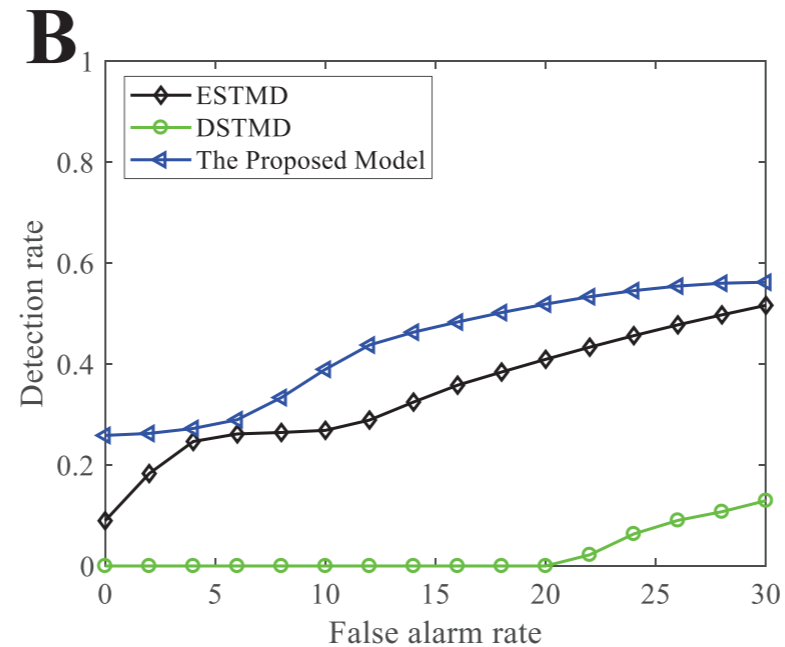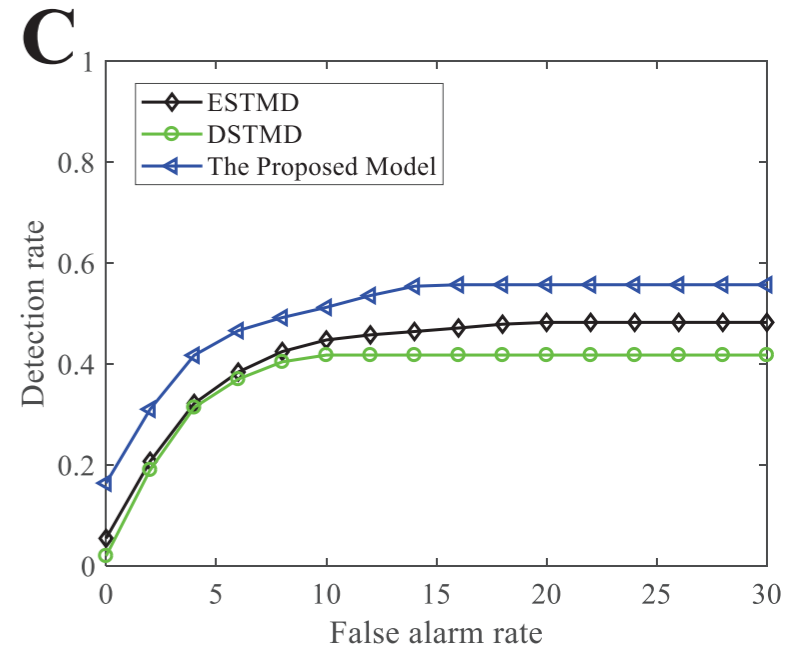

Supplement: Supplementary file 2 [file Data_Sheet_1.ZIP › Supplementary Material Presentation/Fig13-eps-converted-to.pdf]

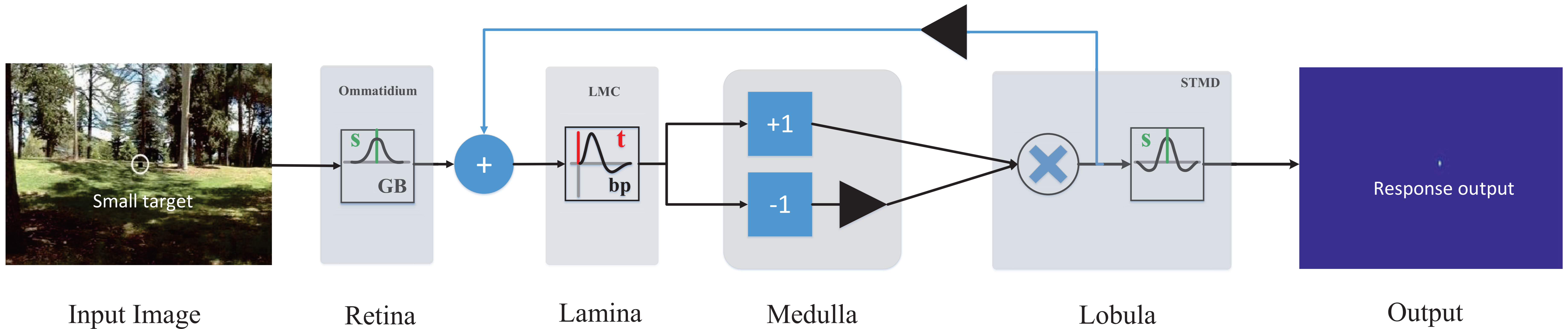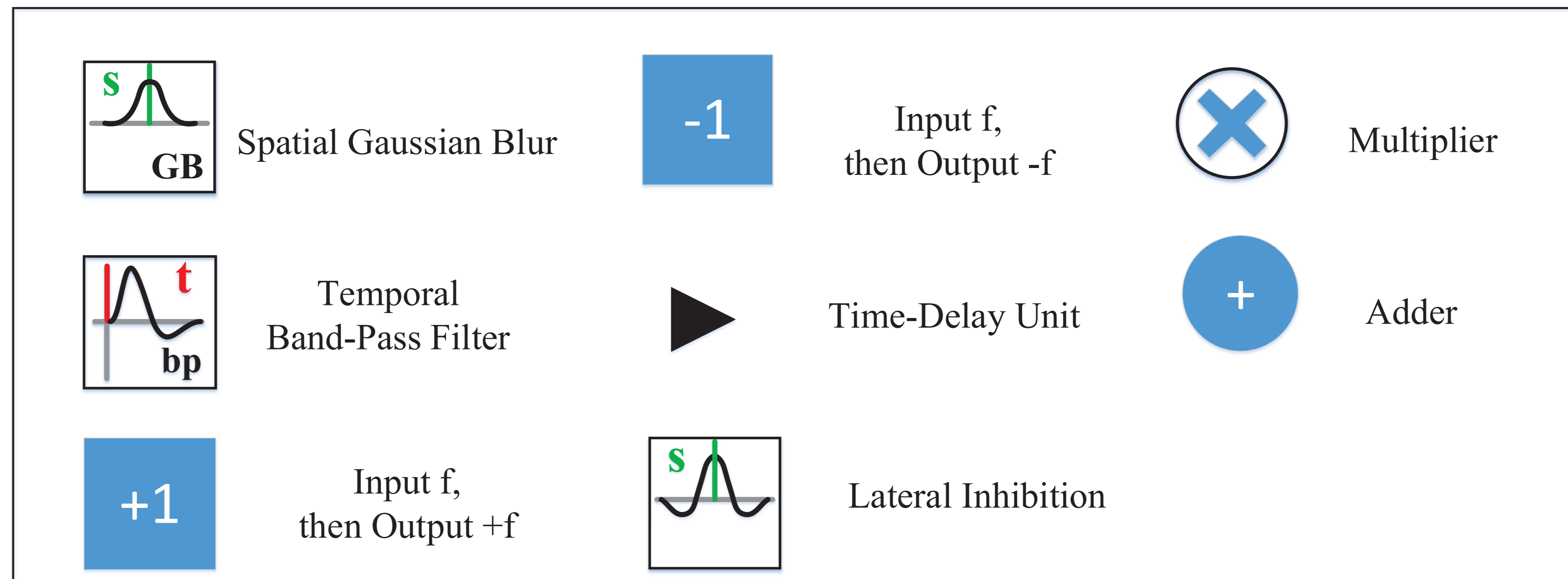

Supplement: Supplementary file 2 [file Data_Sheet_1.ZIP › Supplementary Material Presentation/Fig2-eps-converted-to.pdf]

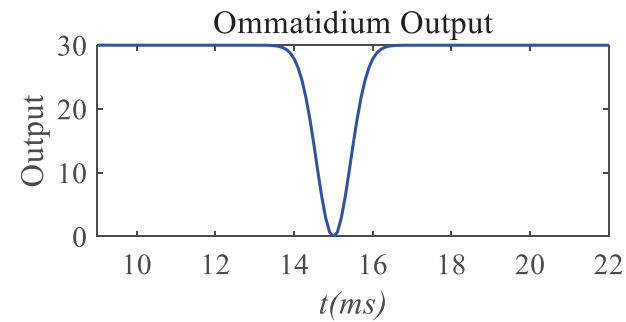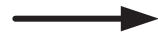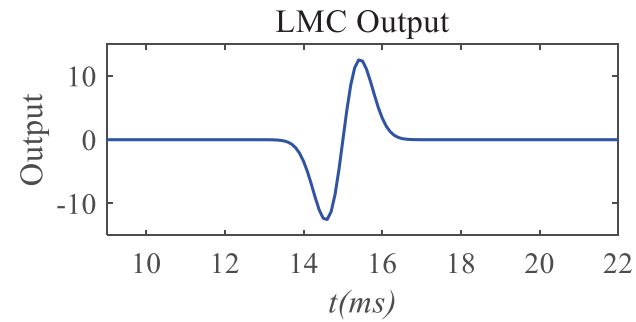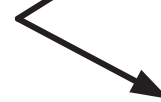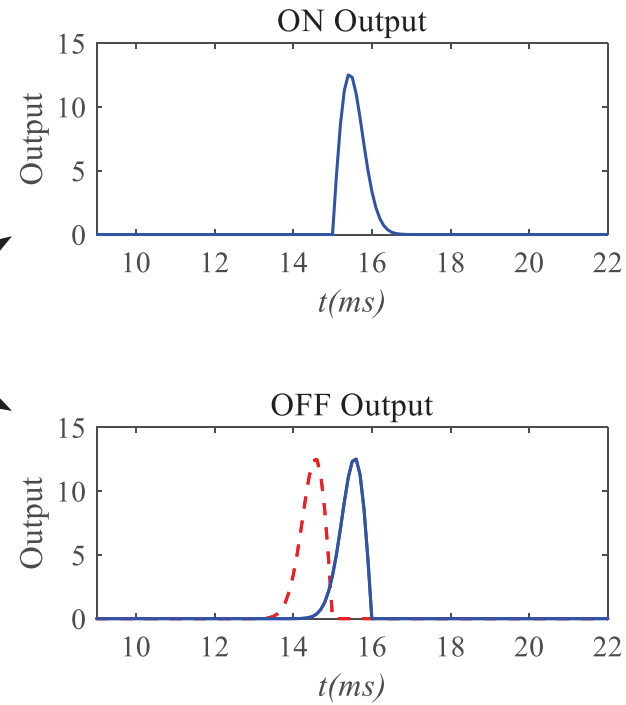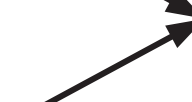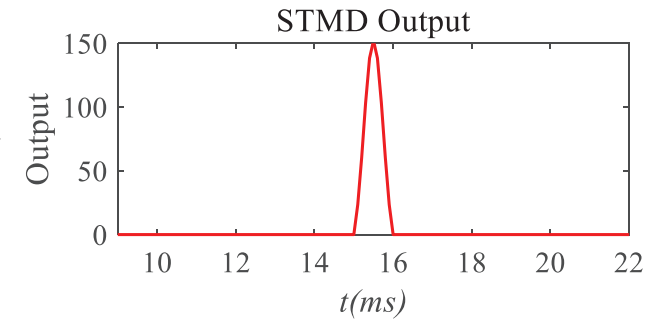

Supplement: Supplementary file 2 [file Data_Sheet_1.ZIP › Supplementary Material Presentation/Fig3-eps-converted-to.pdf]

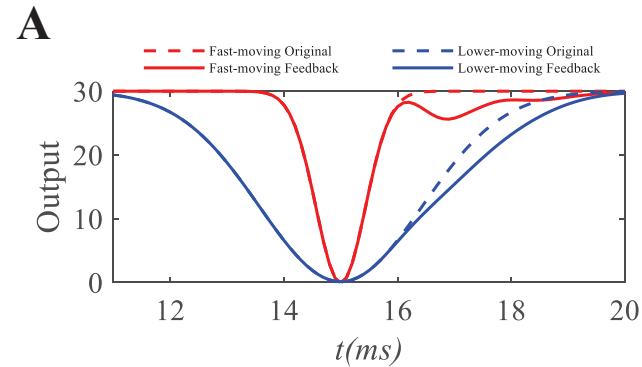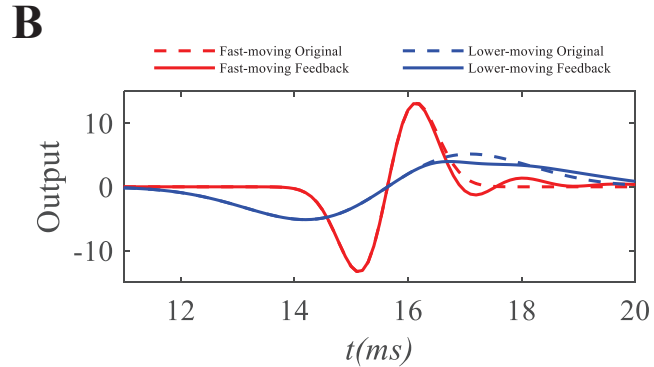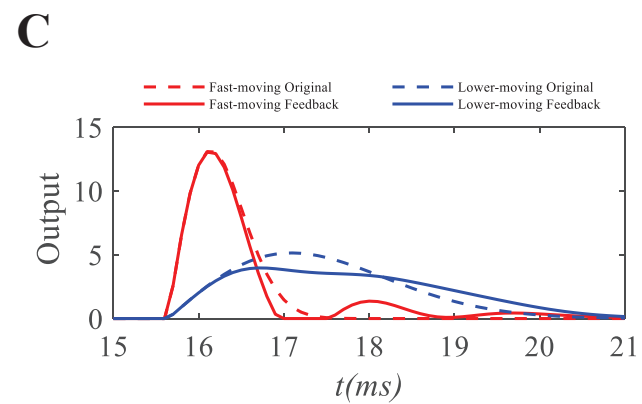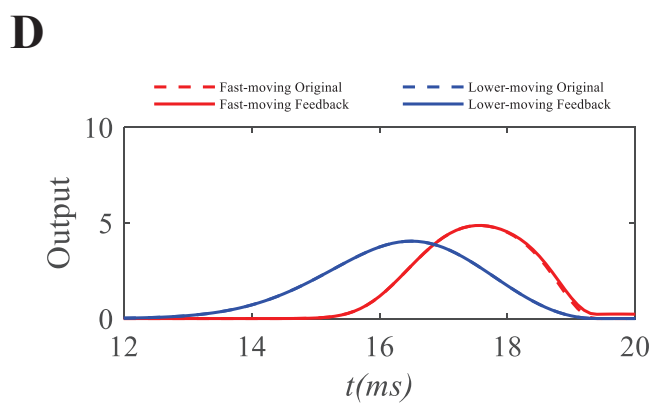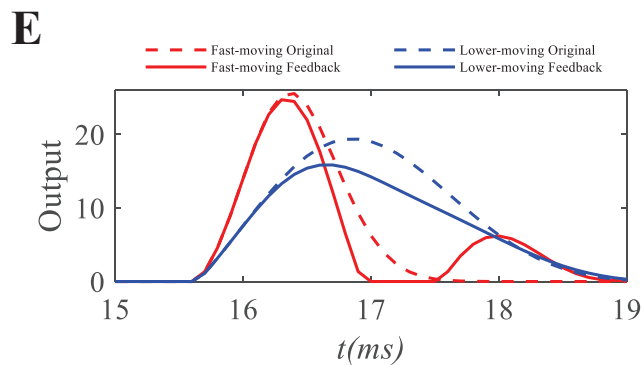

Supplement: Supplementary file 2 [file Data_Sheet_1.ZIP › Supplementary Material Presentation/Fig5-eps-converted-to.pdf]

**A**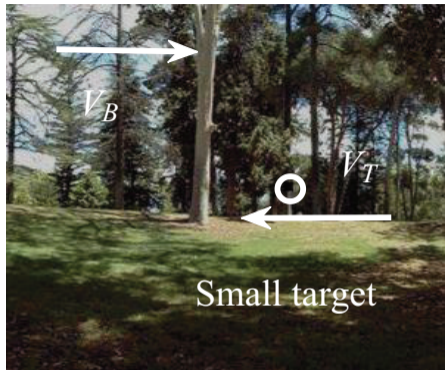

A frame of the initial  
sequence

**B**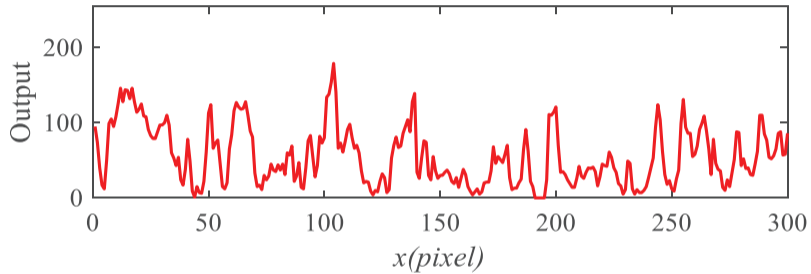

The input signal

Supplement: Supplementary file 2 [file Data_Sheet_1.ZIP › Supplementary Material Presentation/Fig6-eps-converted-to.pdf]

**A**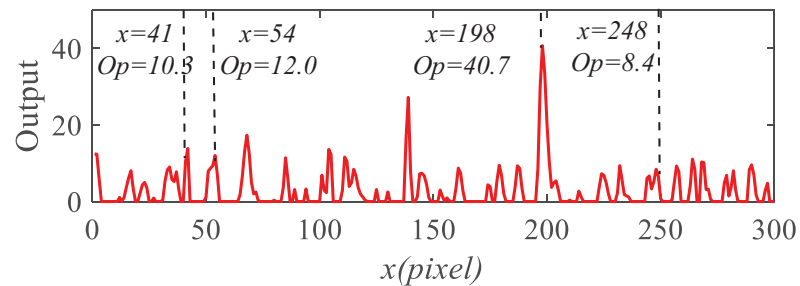

Original ON outputs

**B**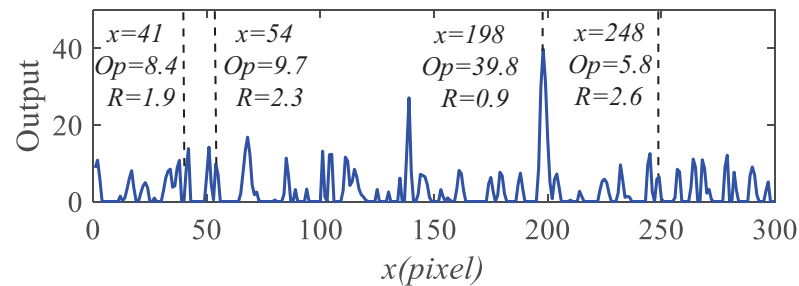

Feedback ON outputs

**C**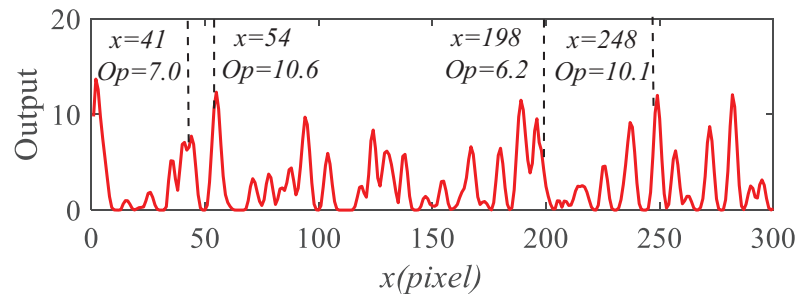

Original OFF outputs

**D**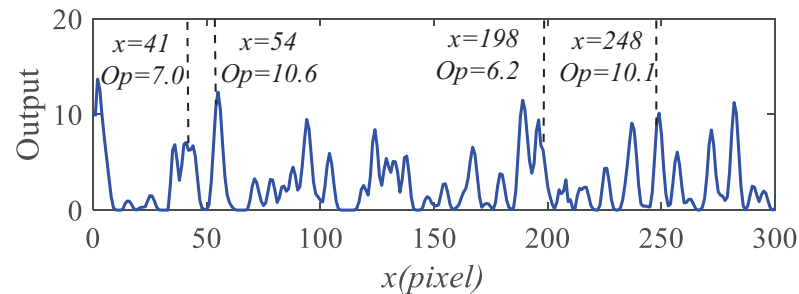

Feedback OFF outputs

Supplement: Supplementary file 2 [file Data_Sheet_1.ZIP › Supplementary Material Presentation/Fig8-eps-converted-to.pdf]

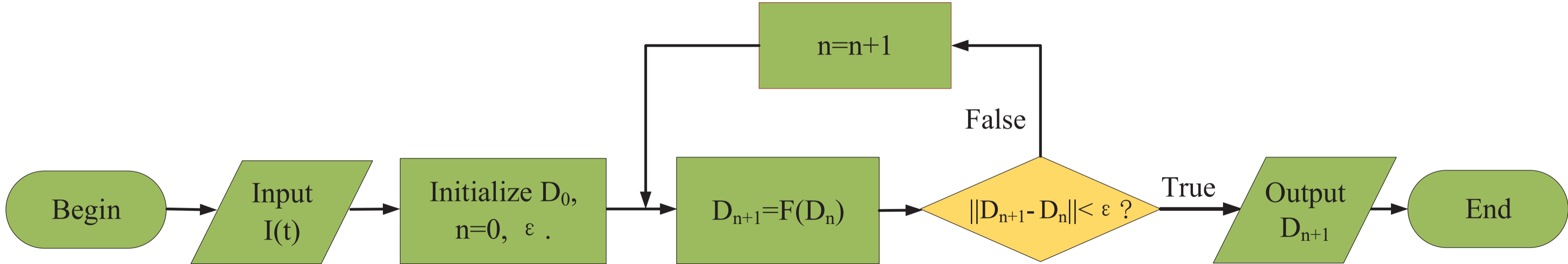

Supplement: Supplementary file 2 [file Data_Sheet_1.ZIP › Supplementary Material Presentation/Fig99-eps-converted-to.pdf]

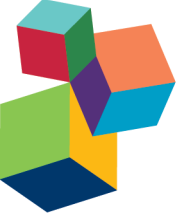

frontiers

Supplement: Supplementary file 2 [file Data_Sheet_1.ZIP › Supplementary Material Presentation/logo1-eps-converted-to.pdf]

A

frontiers  
FOR YOUNG MINDS

B

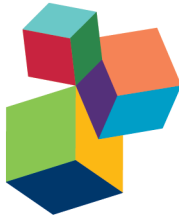

Supplement: Supplementary file 2 [file Data_Sheet_1.ZIP › Supplementary Material Presentation/logos-eps-converted-to.pdf]
